# Characterisation of Quality of Life and Its Utility as a Descriptor of Health Outcomes for People With Profound Intellectual and Multiple Disabilities: A Scoping Review of Primary Studies

**DOI:** 10.1111/jar.70273

**Published:** 2026-07-14

**Authors:** Sarah J. Ballard, Hazel M. Chapman

**Affiliations:** ^1^ Learning Disabilities & Autism Directorate Coventry & Warwickshire NHS Partnership Trust Warwickshire UK; ^2^ University of Chester Chester UK

**Keywords:** health outcomes, profound intellectual disabilities, quality of life, scoping review

## Abstract

**Background:**

Establishing the effectiveness of interventions to promote quality of life is essential to providing evidence‐based care, optimising outcomes and justifying expenditure on such provision. This review explores how quality of life for adults with profound intellectual disabilities is characterised, measured and utilised to evaluate health interventions in the research literature.

**Methods:**

A scoping review of primary research published 2010–2024 was conducted in CINAHL, MEDLINE, APA PsycINFO, APA SocIndex, Education Source, PUBMED, Web of Science and Scopus. 31 publications met inclusion criteria.

**Results:**

Quality of life is multifaceted. No agreed definition or parameters of good, poor, or meaningful changes to quality of life exist for people with profound intellectual disabilities. Existing quality of life scales are not responsive enough to detect changes brought about by health interventions.

**Conclusion:**

More effective tools are needed to provide meaningful quality of life information in relation to health interventions for people with profound intellectual disabilities.

## Lay Summary

- A person's ‘quality of life’ is their view about how closely their life experiences compare to how they would choose them to be.
- It is important that we understand how advice and treatments to keep people healthy affect their quality of life.
- We know there are lots of different things that can affect quality of life, but this can be difficult to measure.
- We need to find better ways to identify and assess the effects of healthcare treatments on quality of life of people with profound intellectual disabilities.

## 1 Introduction

### 1.1 Quality of Life

The World Health Organisation (World Health Organisation 2012) defines quality of life (QOL) as ‘an individual's perception of their position in life in the context of their culture and value systems in which they live and in relation to their goals, expectation, standards and concerns’. The World Health Organisation (WHO) recommended QOL measurement instrument is the WHOQOL, a 100‐question assessment, yielding scores across six domains of living. It is an inherently subjective evaluation of a person's physical, psychological, social, personal and environmental status, scored according to the self‐reported perceptions of the individual concerned. QOL and disability are social constructs, and as such are heavily influenced by culture, governing structures and ideologies, economies, health and social welfare systems (Das 2023). Whilst the WHOQOL has been rigorously tested across many cultures and languages, it does not address how it should be used/interpreted for people who cannot self‐report. Reliance on self‐reporting of QOL is problematic for people who have cognitive or communication impairments, giving rise to an emerging field of health research that aims to address QOL concepts for people who are unable to self‐report them.

Health research about QOL is generally divided into studies developing/validating a measure of QOL, or studies testing the effectiveness of an intervention aiming to improve QOL (Costa Daniel et al. 2021). In this context, definitions and understandings of QOL are extremely varied, from a single overarching concept to a multifaceted one, often driven by the choice of outcome measure where various aspects of well‐being are ‘scored’ (Costa Daniel et al. 2021). These types of measures require the person completing them to assign a numerical value to a series of particular characteristics or activities are known as rating scales. Generally, the rating (or score) indicates a higher or lower level of occurrence or expression for example, 1 = never 2 = sometimes, 3 = often 4 = routinely examples include the San Martin Scale (Verdugo et al. 2013) and the Spanish Personal Outcomes Score (Carbó‐Carreté et al. 2019).

Health‐related quality of life (HRQOL) is a more specific concept and is usually defined in terms of symptom burden/relief in relation to a specific disease or condition (e.g., Multiple Sclerosis Impact scale (Hobart 2001)). As such, HRQOL is generally part of a wider assessment of overall QOL—both being relevant to evaluation of a person's perception of their wellbeing, recognising that a person's experiences are more than their disease or condition. As measures of effectiveness of health interventions, QOL rating scales are problematic as they inherently contain (or fail to account for) additional moderator variables (e.g., social, psychological and environmental determinants) that could influence the outcome (Kovach 2019).

### 1.2 Intellectual Disabilities and Health Inequality

People with intellectual disabilities experience poorer health outcomes, greater risk of dying from preventable causes and reduced access to safe, high‐quality healthcare than the general population, despite the introduction of legislation to promote equity (Ramsey et al. 2022).

People diagnosed with profound intellectual disabilities often experience additional physical disabilities, sensory or mental health difficulties, or behavioural challenges, referred to as ‘profound intellectual and multiple disabilities’ (Heslop et al. 2013; NHS England 2013). People with profound intellectual and multiple disabilities have greater health needs than the general population and those with less severe intellectual disabilities; common manifestations include epilepsy, respiratory, gastro‐intestinal, swallowing, and nutritional disorders, reduced bone density, and postural asymmetry (NHS England 2013; White et al. 2022).

Even with specialist individualised support, people with profound intellectual disabilities generally have an extremely impaired ability to understand, interpret, remember or express information. This means they often lack the mental capacity to make informed choices about their needs, or express whether or not these have been met to their satisfaction (Bellamy et al. 2010). Inevitably, these decisions are taken by others acting as proxies for example, families, carers, healthcare professionals. There is much discussion in the literature about the reliability of proxy reporting, with some studies demonstrating high levels of disagreement between represented persons and their proxies (Lyons et al. 2017). Legislation provides a framework for decisions to be made in the best interests of people who cannot do so for themselves, emphasising the need to consider the impact of a decision on the person's QOL (Guardian OotP 2005). However, defining and describing QOL for people with profound intellectual disabilities is still the subject of much debate.

In England, A key NHS recommendation for people with profound intellectual and multiple disabilities is that holistic care should include specialist physiotherapy to manage pain and distress, protect body shape, and promote of health and wellbeing (Mansell 2010; Doukas et al. 2017). Specialist Physiotherapists prescribe 24‐h postural care interventions and equipment that protect and maintain body shape, function and QOL (Bruce and Standley 2019). However, no guidance is provided about how to assess QOL, or how recommended postural care interventions should be evaluated in terms of effectiveness for promoting QOL.

### 1.3 Resources and Health Provision

The NHS has finite resources, with clinicians increasingly required to maximise health whilst minimising expenditure (Freudenberg 2021). Unsurprisingly, the most common reason for non‐provision of postural management equipment is lack of funding (Public Health England 2018), despite a legislative duty for free provision of specialist equipment by the NHS (Oliver 2006) and Local Authorities (Clements 2014). However, there is also legislative imperative to provide only the most cost‐effective option, and deciding what is cost effective in terms of QOL is difficult. Without sufficient research evidence or agreed QOL outcome criteria against which such provision can be measured, it is impossible to robustly demonstrate clinical and cost effectiveness. It is therefore inevitable that NHS and local authorities will use their ultimate discretion to prioritise lower‐cost interventions (Mandelstam 2016). Often, families and charities are compelled to make up the shortfall, or the person must endure without (NHS Improving Quality 2014). Thus, a dichotomy is created between the stated aims of NHS provision for people with profound intellectual and multiple disabilities, and what is actually provided.

Pragmatically, an agreed definition of what constitutes a positive health or QOL outcome for a person with profound intellectual disabilities is required before ways to demonstrate or predict such outcomes for specific interventions can be developed. Equally, definition and demonstration of outcomes is only useful if it works in practice to support such provision (Kovach 2019).

### 1.4 Aims

1. To identify and critically evaluate how QOL for adults with profound intellectual disabilities is characterised in the research literature.
2. To examine and describe methods used in contemporary research to measure QOL for adults with profound intellectual disabilities.
3. To explore how researchers use QOL measures to describe health and wellbeing outcomes for adults with profound intellectual disabilities.
4. To identify areas of strength and weakness in the research evidence, including areas of controversy and gaps in knowledge that may indicate the need for further research.

## 2 Method

This literature review was conducted following Arksey and O'Malley's framework (Arksey and O'Malley 2005), to identify and select relevant studies, chart, collate and report the results.

### 2.1 Identification and Eligibility of Sources

All types of primary quantitative, qualitative and mixed methods research were considered for inclusion in the review. Literature reviews, summaries, policy and opinion articles were not included since the full text of research included in these articles is not available for data extraction and critical evaluation. However, the reference lists of these articles were searched for further primary studies. 2010 was chosen as the earliest inclusion date to encompass research undertaken since (and immediately prior to) the publication of the CIPOLD report (2013) (Heslop et al. 2013), which provided accessible national data about the health and QOL inequalities experienced by people with profound intellectual disabilities for the first time.

### 2.2 Study Selection—Keywords and Search Terms

Research concerning QOL is often qualitative or part‐qualitative in nature, so keywords and their synonyms were identified using the SPIDER tool, as this captures qualitative literature more effectively than the PICO tool (Cooke et al. 2012; Methley et al. 2014). Medical subject headings (MESH) were searched in CINAHL and PUBMED databases to identify further synonyms and search phrases. Boolean operators and truncations were identified to create search strings. These were evaluated and refined using test searches. Key words and search terms are shown in Table 2.

Searches were carried out iteratively, with the last search on 31/12/24 in the following databases: CINAHL, MEDLINE, APA PsycINFO, APA SocIndex, Education Source, PUBMED, Web of Science and Scopus. Hand searching through reference lists of returned articles was also undertaken.

### 2.3 Search Strategy

Searches were conducted iteratively combining search strings applied to ‘all fields’ or ‘all text’ depending on the database as shown in Table 3.

A total of 2, 971 returned article titles and abstracts were screened for eligibility by the first author. Those which met the criteria (277) were screened for duplicates (110), which were then removed. The full texts of the remaining 167 articles were retrieved and assessed for eligibility against inclusion/exclusion criteria (Table 1).

**TABLE 1:** Inclusion and exclusion criteria.

Inclusion criteria	Exclusion criteria
‐ Participants are adults (18 years+) with severe or profound intellectual and multiple disabilities or their proxies	‐ Child only studies (age < 18 years)
‐Total quality of life, or individual facet of quality of life (as described in the WHO definition) is being described/evaluated	‐ Participants have mild, moderate or no intellectual disabilities
	‐ Not assessing quality of life
‐ Published between January 2010 and May 2026	‐ Quality of life of the *proxies only* is being described/evaluated
‐ Peer reviewed research	‐ Not primary research
‐ English language	‐ Language not English

**TABLE 2:** Keywords and search terms.

Key words and search terms					
Search topic	How is quality of life assessed, measured or described for adults with profound intellectual disabilities and physical disabilities?				
SPIDER	Sample	Phenomenon of interest	Design	Evaluation	Research type
Keywords	Profound intellectual and multiple disabilities	Quality of life	Interview	Efficacy	Quantitative
	PIMD	Health related quality of life	Focus Group	Effectiveness	Qualitative
	Profound and multiple learning disabilities	Well being	Survey	Impact	Mixed methods
	PMLD	Well‐being	Questionnaire	Outcome measure	
	Profound Intellectual disabilities		Case study	Outcome assessment	
	Physical disabilities		Case series	Outcome tool	
	Motor impairment		Trial		
Boolean search terms	‘Profound intellectual and multiple disabilities’ OR ‘Profound and multiple learning disabilities’ OR ‘profound intellectual and physical disabilities’ OR ‘Profound intellectual disabilities and severe motor impairment’	‘Quality of life OR Well being OR Well‐being OR health related quality of life’	Interview OR Focus Group OR Survey OR Questionnaire	Efficacy OR Effect* OR	Quant* OR Qualitat* OR ‘Mixed Methods’
				Impact OR	
				Outcome measures OR Outcome assessment OR Outcome tool	
			OR Case Study		
			Or Case Series		
			OR Trial		

**TABLE 3:** Search strategy and articles returned.

Search terms with Boolean operators	‘Profound intellectual and multiple disabilities’ OR ‘Profound and multiple learning disabilities’ OR ‘profound intellectual and physical disabilities’ OR ‘Profound intellectual disabilities and severe motor impairment’ AND ‘Quality of life OR health status OR well being OR well‐being OR health related quality of life OR outcome measures OR outcome assessment OR outcome tool’				
Limiters	All text (‘TX’) English, 2010–2026, all adult				
Date(s)	Various, August–December 2024, updated May 2026				
Database	Number of articles returned	Removed on abstract screen	Number remaining	Duplicates removed	Number remaining to be screened further
CINAHL	517	473	44	—	44
MEDLINE	217	186	31	14	17
PsycINFO	184	154	29	13	16
SocIndex	195	187	8	2	6
Education Source	377	360	17	7	10
PUBMED	176	140	36	23	13
Scopus	773	733	40	16	24
Web of science	508	460	48	35	13
Hand search	24	—	24	—	24
Total	2, 971	2693	277	110	167

**TABLE 4:** Themes and subthemes.

Themes		Associated code group (see Table A3)	Sub‐themes
1	How QOL for people with profound intellectual and multiple disabilities is characterised	Descriptors and indicators	Domains of WHOQOL
			Numerical and narrative
			Use of language
2	How QOL for people with profound intellectual and multiple disabilities is assessed/measured	Methods and tools	Physiological measures
			Rating scales
			Proxies and tacit knowledge
3	How QOL measures are utilised to demonstrate health/wellbeing outcomes for people with profound intellectual and multiple disabilities	Outcome measurement	Validity, reliability and
			Responsiveness
			QOL outcomes and thresholds

**TABLE 5:** Methods of assessment of QOL.

Method	Area(s) of focus	Studies using this method
Rating Scales		
Family Quality Of Life Survey (FQOLS)	Whole family—health, relationships, support, finances, services support, leisure and community, values	Bertelli et al. (2011, 2019)
Quality Of Life In PMD (QOLIP)	Being, becoming, belonging	Bertelli et al. (2011, 2019)
Behaviour Assessment Scale (BAS)	Behaviour of the individual to communicate distress or contentment.	Bossink et al. (2017)
Personal Outcomes Score (POS)	Personal development, social inclusion, participation, physical wellbeing, emotional wellbeing, material wellbeing, rights, interpersonal relations, independence, self‐determination,	Carbó‐Carreté et al. (2019)
		Tatsuta et al. (2025)
		Neveu et al. (2025)
International Physical Activity Questionnaire (IPAQ)	Evaluation of physical activity levels for health related QOL	Dairo et al. (2017)
Quality of Life Profound Multiple Disability (QOL‐PMD)	Physical wellbeing, social wellbeing, communication and influence, development, activities	Bossink et al. (2017)
		de‐Gues Neelen et al. (2019)
San Martin Scale	Self‐determination, emotional wellbeing, material wellbeing, rights, personal development, social inclusion, interpersonal relations	Cameranesi et al. (2022)
		Gómez et al. (2015)
		Verdugo et al. (2013)
		Navas et al. (2025)
		Herps et al. (2016)
Mood Interest and Pleasure Questionnaire (MIPQ)	Mood, pleasure, interest	Vos et al. (2013b, 2010a)
Resident Choice Scale (RCS)	Opportunities and capacity to make choices about their home environment, routines, activities and support structures	Navas et al. (2025, 2024)
Active Support Participation Measure (ASPM)	Level of support required to engage with activities of daily living	Navas et al. (2025, 2024)
CPADULT	Activities of daily living, positioning transferring and mobility, comfort and emotions, communication and social interactions, health, overall QOL	Zalmstra et al. (2023)
		Zalmstra et al. (2021)
Pain Behaviours Assessment Tool	Presence or absence of behaviours associated with pain	Enninga et al. (2023)
Vineland Adaptive Behaviour Scale	Receptive and expressive communication, personal and domestic life, socialisation, play and leisure, motor skills, community.	Neveu et al. (2025)
Brunet Lezine Scale	Neurodevelopmental status	Baumstarck et al. (2025)
Functional Independence Measure	Level of support required to participate in functional activities	Baumstarck et al. (2025)
Gross Motor Function Classification Scale	Motor function in relation to ambulation and mobility	Baumstarck et al. (2025)
POLYQoL	Quality of life for people with multiple disabilities	Baumstarck et al. (2025)
Physiological measures		
Pulse oximetry	Alertness, emotional state, cardiovascular fitness, oxygen levels	Bossink et al. (2017)
Modified Ashworth Scale	Muscle tone, relaxation	Bossink et al. (2017)
Body Mass Index	Height: weight ratio	Bossink et al. (2017)
Skin Conductance	Electrical activity in the skin in relation to emotion‐induced sweating	Vos et al. (2010)
Movement	Emotional state	Vos et al. (2013b)
Heart rate	Emotional state, cardiovascular fitness	Vos et al. (2013b, 2010a)
		Bossink et al. (2017)
Respiration rate	Emotional state, cardiovascular fitness	Vos et al. (2013b, 2010a)
		Bossink et al. (2017)
Others		
Time engaged in activity	Opportunities for meaningful activities	Beadle‐Brown et al. (2016)
Number and quality of goals set	Opportunities for meaningful activities	Herps et al. (2016)
Tacit knowledge	Implicitly understanding a person's needs	Nieuwenhuijse et al. (2020, 2022)
Subjective description	Describing a person's needs and whether they are met	Nieuwenhuijse et al. (2020)
		Nieuwenhuijse et al. (2022)
		Matérne and Holmefur (2022)
		Talman et al. (2019)
Observed behaviour	Observing a person's behaviour in relation to their needs being met or unmet	Talman et al. (2021)
		Vos et al. (2010a)
Number of medical devices	Eg. mechanical ventilation, gastrostomy, tracheostomy	Baumstarck et al. (2025)
Number of medications	Eg. laxatives, anti‐convulsants, analgesics, psychotropics	Baumstarck et al. (2025)

### 2.4 Data Extraction and Critical Appraisal

All eligible articles were critically appraised using the Mixed Methods Appraisal Tool (MMAT) (Hong et al. 2018; Pace et al. 2012). and then their data systematically extracted into tables (Tables A1 and A2). A score was assigned to each study based on how well the MMAT quality criteria were met. Fully met criteria scored 2, partially met 1, and unmet 0. Scoring was used to indicate overall quality alongside narrative critical evaluation and is displayed alongside the extracted data. No articles were excluded on the basis of quality (Hong et al. 2018).

### 2.5 Data Coding and Analysis

The primary findings from each paper were coded (open coding) by SB according to their underlying concepts and synonyms, following the method established by (Coughlan and Cronin 2021) p115–116. Selected papers were read and coding samples were checked by HMC. Where quantitative scales were used, the actual numbers were rarely reported, so they were coded by researchers' interpretations. A total of 84 codes were assigned to concepts reflecting one aspect of QOL assessment for people with profound intellectual disabilities described in the studies. Themes were created by grouping codes with similar meanings and their associated texts then assigning headings to reflect the contents.

Labels were also assigned to each outcome tool/method of assessment to enable comparison between papers. Codes were then refined and arranged into themes (see Table A3) for overall analysis. Separately, codes were grouped by aligning them to each facet of the WHOQOL and arranged into the 6 corresponding domains (Table A4) for comparison and analysis. An Excel spreadsheet was used to generate charts and graphic representations of the data.

## 3 Results

**FIGURE 1:**
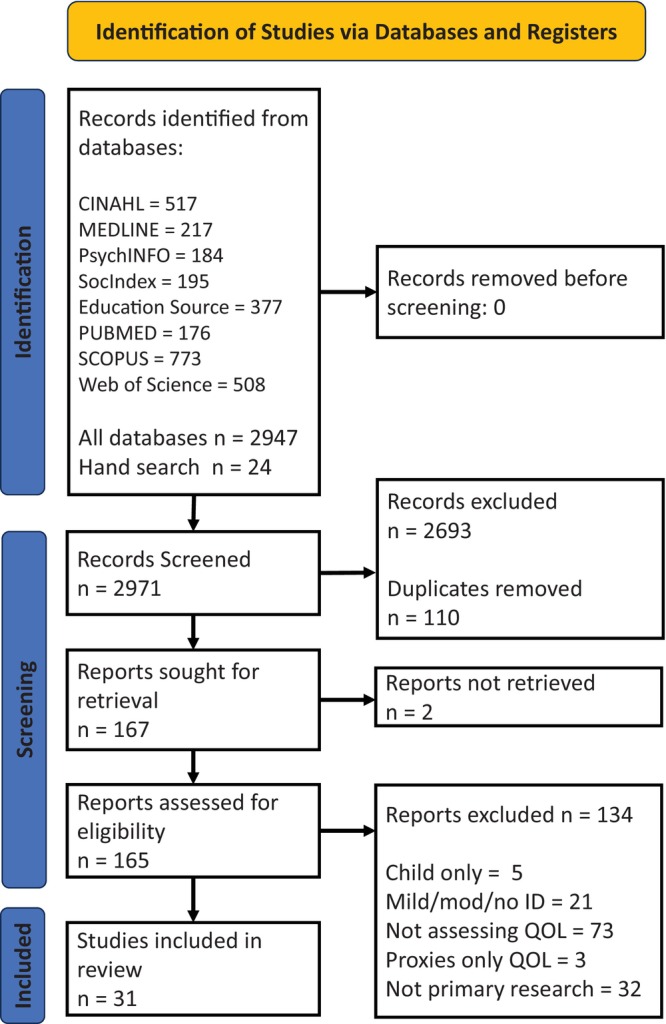
PRISMA DIAGRAM adapted from Page, McKenzie (Page et al. 2021).

A total of 2971 articles were screened for eligibility by title and abstract, with 167 articles meeting criteria for full text appraisal. 167 full text articles were retrieved and assessed for eligibility, with 31 articles being included in the review (see Figure 1: PRISMA diagram). Articles were mostly excluded on the basis that they were secondary research or were not assessing QOL.

### 3.1 Study Design & Quality

18 studies were quantitative designs with observational and cross‐sectional study designs being the most prevalent. 8 studies were qualitative and 5 mixed methods, with semi‐structured interviews the dominant method of data collection. Overall quality of studies was moderate to good, with the lowest MMAT score being 5/10 (range 5–9). Strengths included appropriately sized, inclusive, representative samples in real‐life contexts. Limitations included insufficient justification for methods, inadequate explanation of confounding factors, and interpretations of findings that were only partially substantiated by the data.

### 3.2 Participant Characteristics

A total of 5716 participants were included in the review, of whom 3098 were adults with severe or profound intellectual disabilities aged 18–85 years (56% male, 45% female). A further 1312 people with an unclear age or level of intellectual disabilities were also included. The remaining 1306 participants were proxies, of whom 1018 (77.9%) were care staff, 237 (18.1%) family members, and 51 (3.9%) expert care professionals. Sample size ranged from 4 to 1770 participants.

People with intellectual disabilities participated exclusively in community settings. Studies conducted in academic or hospital settings were with expert professionals only. Participant characteristics by study are summarised in Table A1.

### 3.3 Location

Two studies were conducted in Canada, with the remainder in Europe: Netherlands (Heslop et al. 2013), Spain (Carbó‐Carreté et al. 2019), Belgium (Carbó‐Carreté et al. 2019), Sweden (Costa Daniel et al. 2021), Italy (Das 2023), Denmark (World Health Organisation 2012), France (World Health Organisation 2012) and the UK (Costa Daniel et al. 2021). Studies completed within the same country tended to share one or more authors—meaning that there are few researchers working in this field. All included studies were conducted in ‘developed’ countries but varied considerably in terms of geopolitical, health, and social care structures and processes. It is accepted that these factors influence social constructs of disabilities, health, and QOL (Das 2023) meaning that direct comparison should be approached cautiously. Further, it reflects the dearth of research data in this field outside mainland Europe.

### 3.4 Theoretical Positionality

Most studies (*n* = 22) collected quantitative data in the form of a ‘score’ or ‘rating’ defined by a specific tool, or observational data which was then coded/categorised and represented quantitatively. Some studies also collected qualitative data and then coded/categorised this in a quantitative way. Overall, a quantitative presentation of results was favoured in an attempt to represent a single reality of QOL for people with profound intellectual disabilities, consistent with post‐positivist ideology, despite 5 studies citing a pragmatist approach. Five studies employed interpretive phenomenology as their underpinning theoretical approach to explore the perceptions of: parents (Nieuwenhuijse, Willems, van Goudoever, and Olsman 2023); physicians (Nieuwenhuijse et al. 2020); professional caregivers (Nieuwenhuijse et al. 2022; Matérne and Holmefur 2022) and people with intellectual disabilities (Talman et al. 2019). One study (Talman et al. 2021) used descriptive phenomenology to explore the experiences of people with intellectual disabilities.

### 3.5 Ethics

All studies gained appropriate ethical approval according to local legislation and requirements. It is worth noting that practices described in some studies (such as parental consent for adult children) would not be permissible under UK law. There was acknowledgment that research activities could cause transient distress, particularly those observing responses to pain and unpleasant stimuli, and steps were taken to alleviate these where practicable (Enninga et al. 2023; Vos et al. 2013a, 2013b).

## 4 Findings

The following themes and sub‐themes were found from the literature (Table 4).

### 4.1 Theme 1: How QOL Is Characterised

#### 4.1.1 Descriptors and Indicators in Relation to WHOQOL Domains

Overall, authors gave greatest prominence to the ‘physical’ (21 studies), ‘psychological’ (20 studies) and ‘social’ (17 studies) domains of WHOQOL. The most mentioned descriptors of QOL were ‘emotional wellbeing’ and ‘physical wellbeing’ (15 and 14 citations respectively), followed by ‘social interactions and belonging’ and ‘environmental’ indicators (13 citations). 10 studies cited ‘independence’. Least reported indicators were ‘sexual fulfilment’ (1 citation) and ‘values’ (2 citations).

**FIGURE 2:**
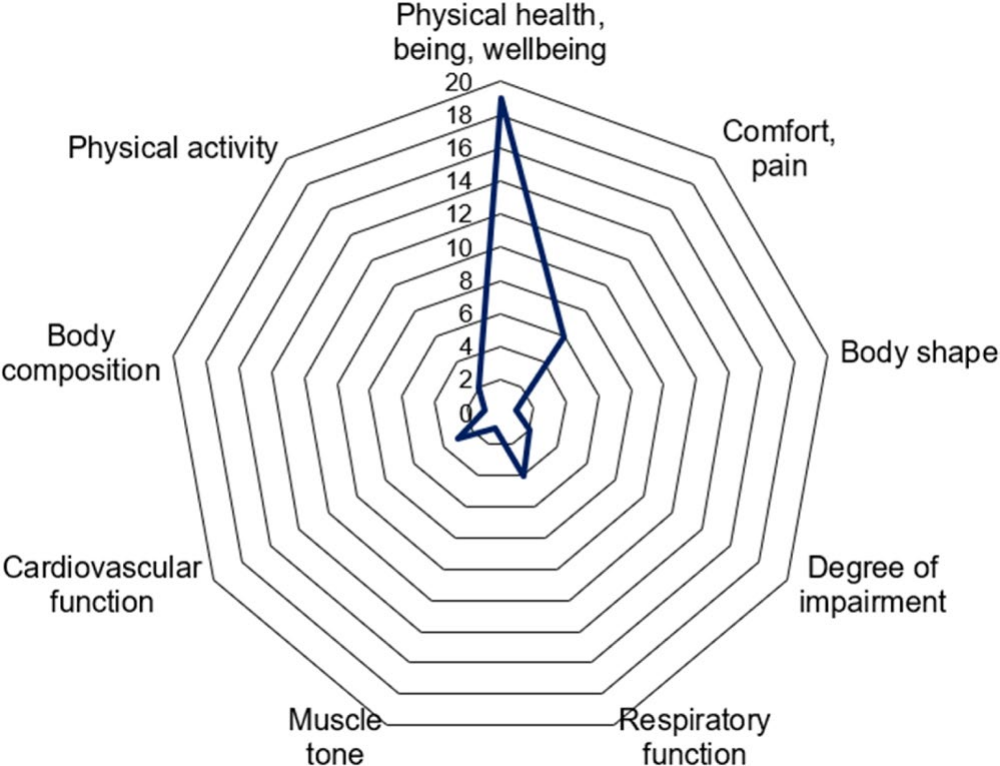
No. of studies citing concepts relevant to the physical capacity domain of the WHOQOL.

**FIGURE 3:**
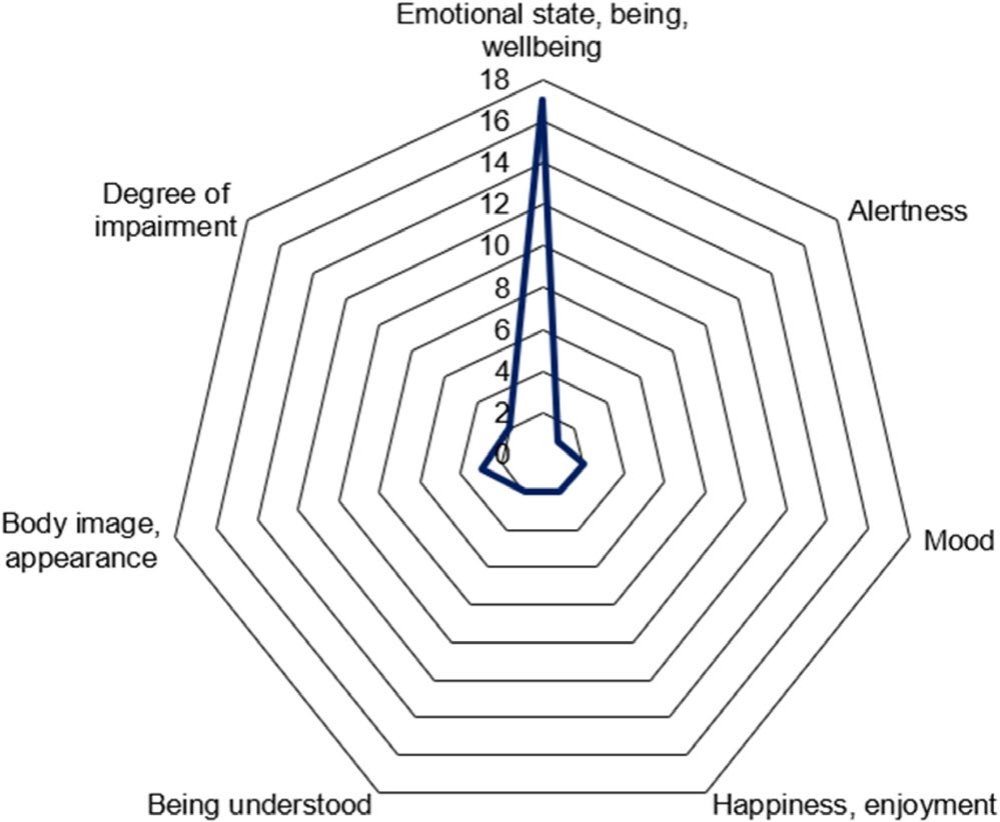
No. of studies citing concepts relevant to the psychological domain of the WHOQOL.

**FIGURE 4:**
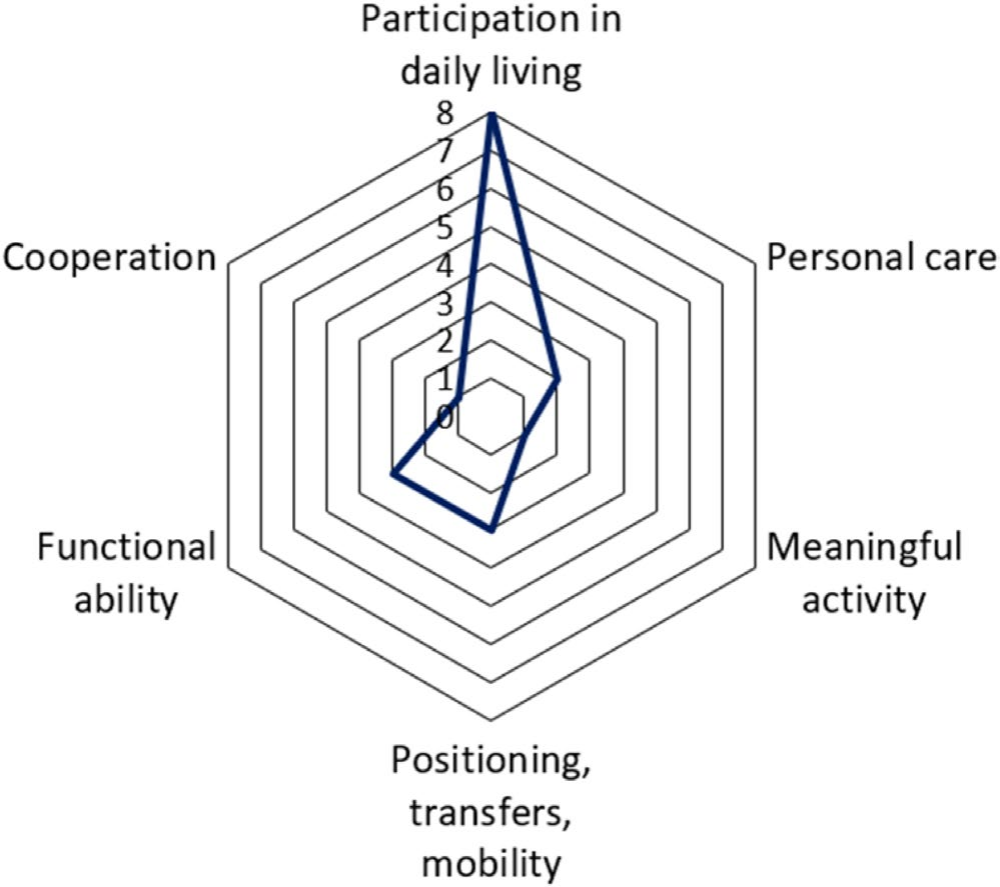
No. of studies citing concepts relevant to the independence domain of the WHOQOL.

**FIGURE 5:**
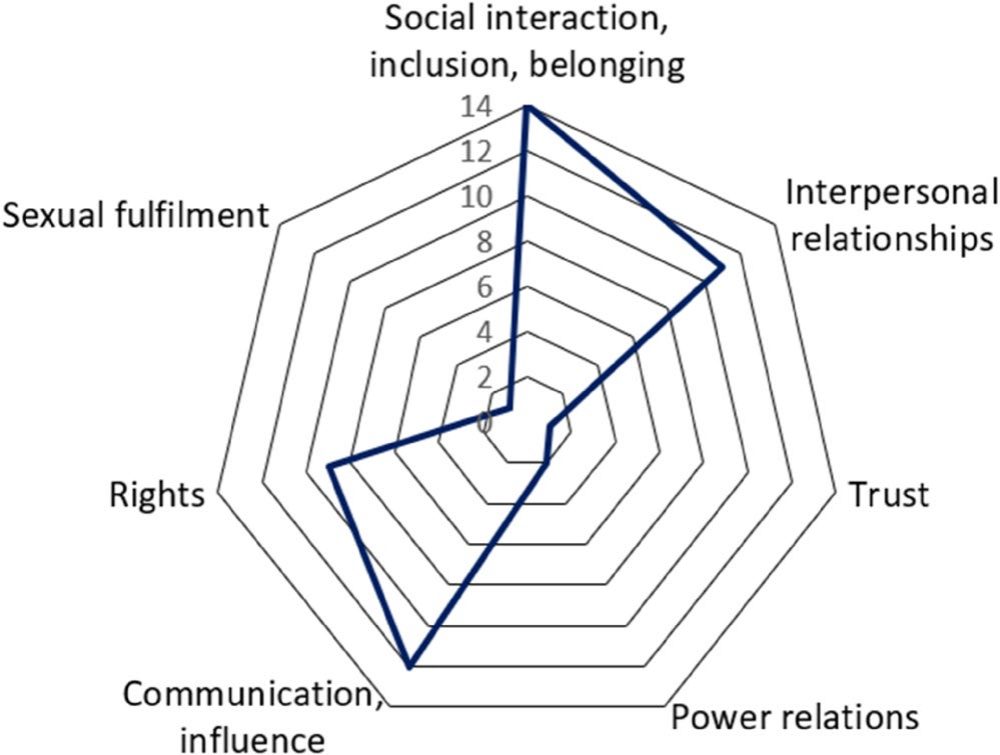
No. of studies citing concepts relevant to the social relationships domain of the WHOQOL.

**FIGURE 6:**
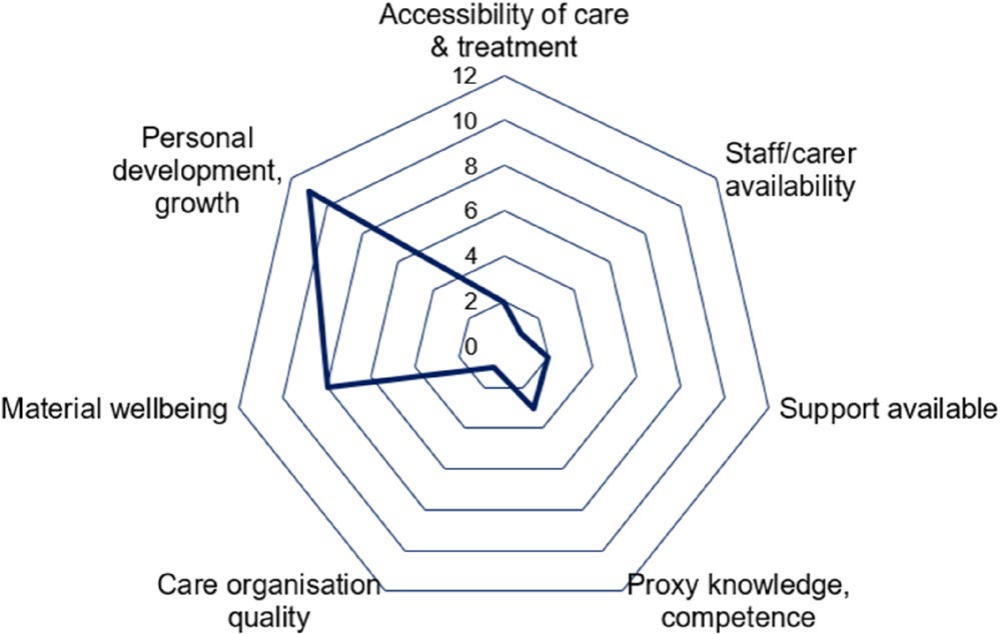
No. of studies citing concepts relevant to the environment domain of the WHOQOL.

Figures 2, 3, 4, 5, 6 show the number of papers that cited each concept, and how these align with the first 5 domains of the WHOQOL. The 6th domain (spiritual, religion, personal beliefs) is not shown graphically, as only 1 descriptor ‘values’ was cited in 2 papers by the same authors (Bertelli et al. 2011, 2019).

The physical capacity domain is dominated by the concept of physical health, with relatively infrequent mention of pain and comfort, and other indicators (Figure 2).

Emotional state was the most reported concept allied to the psychological domain of the WHOQOL (Figure 3). Other factors such as being understood, body image, and happiness were each cited by only one paper.

Participation in activities of daily living was the most reported feature of the independence domain, including activities such as washing, dressing, eating and drinking. Being able to engage in meaningful activities outside of the necessities of daily living was cited by only one paper (Figure 4).

The closely related concepts of social interaction and interpersonal relationships were reported as being highly influential on a person's QOL (Figure 5), The facets of trust, rights, power relations and sexual fulfilment were rarely cited.

Having the facility to undertake personal development and growth, and material wellbeing were the most frequently cited environmental factors. Affecting QOL (Figure 6).

#### 4.1.2 Numerical and Narrative Expressions

Only 7 studies (Nieuwenhuijse, Willems, van Goudoever, and Olsman 2023; Nieuwenhuijse et al. 2020, 2022; Matérne and Holmefur 2022; Talman et al. 2019, 2021; Bradshaw et al. 2024) allowed participants the freedom to describe QOL and its possible meanings for people with profound intellectual disabilities, without requiring a level of agreement with a pre‐existing scale/measure. These studies described ‘good/better’ and ‘poor/worse’ QOL in terms of emotional, physical, psychological, environmental, and care quality.

Good QOL was described in terms of: happiness; pleasure; enjoyment; relaxation; social contact and relationships; being understood; safety; absence of pain and illness; absence of seizure activity; absence of difficulty eating or breathing (Nieuwenhuijse et al. 2020, 2022; Nieuwenhuijse, Willems, van Goudoever, and Olsman 2023). Additional indicators of good QOL were being: listened to; supported to express views; able to participate in daily life; involved in decision making; communicated with effectively; enabled to share power and responsibility (Talman et al. 2019, 2021). One study (Matérne and Holmefur 2022) described QOL as being directly linked to quality of care, asserting QOL is determined by the knowledge, skills, and availability of staff to advocate for and enable participation through individually tailored support, adaptation and responsiveness. Bradshaw et al. (Bradshaw et al. 2024) describe factors associated with ‘better’ QOL, including consistency, opportunities for social contact, access to health and social services and meaningful activities.

Poor QOL was generally described as the absence of factors required to be present, or presence of those required to be absent for good QOL. Additional indicators of excessive sleeping, suffering, tense muscles, choking, and burden too great for family were also described as contributing to poor QOL (Nieuwenhuijse et al. 2020; Olsman et al. 2021). (Bradshaw et al. 2024). specifically mention deterioration in body shape (linked to poor posture) as negatively affecting QOL. Institutionalisation was identified as a significant contributor to poor QOL by 2 studies (Cameranesi et al. 2022; Navas et al. 2024). There were suggestions that a person could have a good QOL if there were sufficient positive factors to balance the negative ones (Nieuwenhuijse et al. 2020; Vos et al. 2010a).

#### 4.1.3 Use of Language

Some measures were developed or used in a language other than English for example The Personal Outcome Scale (Carbó‐Carreté et al. 2019; Tatsuta et al. 2025; Neveu et al. 2025), so interpretation is subject to the quality of translation in cultural context. Additionally, some measures easily yield a Likert scale response (always, often, sometimes, never) for example, San Martin Scale (Verdugo et al. 2013) The language in other scales is more nuanced and person‐centred requiring evaluation in relation to the needs and abilities of the specific individual for example, QOL‐PMD (Palisano 1997)^.^

### 4.2 Theme 2: How QOL for People With Profound Intellectual and Multiple Disabilities Is Assessed/Measured

#### 4.2.1 Methods of Assessment of QOL

**FIGURE A6:**
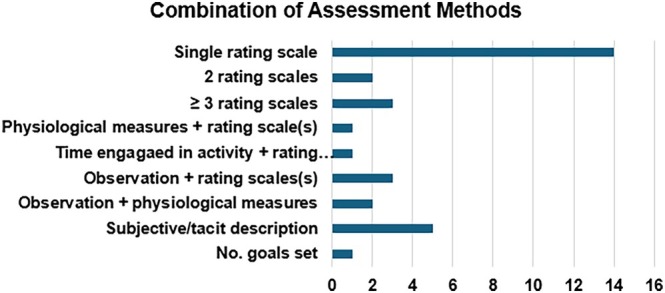
Combination of assessment methods of QOL.

In all, 31 different methods were employed broadly grouped into rating scales, physiological measures and others, each with different areas of focus (Table 5). 14 studies used a single rating scale or tool to assess QOL; the remaining studies used different combinations of tools and methods (see Table A5 and Figure A6).

#### 4.2.2 Rating Scales

None of the rating scales used in these studies encompassed all 6 domains/24 facets of the WHOQOL. Only 2 had a concept pertinent to the spiritual/religion/personal beliefs domain, and none contributed to the facts of work capacity, physical environment/climate or transport. The scales that covered most facets of the WHOQOL were the QOL‐PMD (Doukas et al. 2017) and San Martin Scale (Lyons et al. 2017). Table A7 shows how different aspects of the rating scales used in the studies align with the WHOQOL domains and facets.

The WHOQOL domains most included in the rating scales were ‘participation in and opportunities for recreation/leisure activities’ (8 scales), ‘personal relationships’ (8 scales), and ‘activities of daily living’ (7 scales), consistent with the components of QOL described as most important in the qualitative studies (Nieuwenhuijse, Willems, van Goudoever, and Olsman 2023; Nieuwenhuijse et al. 2020, 2022; Matérne and Holmefur 2022; Talman et al. 2019, 2021).

#### 4.2.3 Physiological Measures

Even where studies utilised similar measures, there was disagreement about how these should be interpreted. Bossink et al. (Bossink et al. 2017) considered QOL as being separate from physiological measures, noting that oxygen saturation improved post intervention, whereas QOL score (and other physiological measures) did not. By contrast, Vos et al. (Vos et al. 2013a, 2013b, 2010a, 2010b) sought to use physiological measures as indicators of emotional wellbeing and QOL by demonstrating congruence between physiological responses and emotional behaviours.

The use of automated devices such as accelerometers (Vos et al. 2013a) (detects movement), pulse oximeters (Vos et al. 2013a, 2010a; Bossink et al. 2017) (measures heart rate and blood oxygen saturation), and skin conductance sensors (Vos et al. 2010b) (measures electrical changes in the skin caused by sweat gland activity) was reliable but not always well tolerated by participants.

#### 4.2.4 Proxies & Tacit Knowledge

**FIGURE 7:**
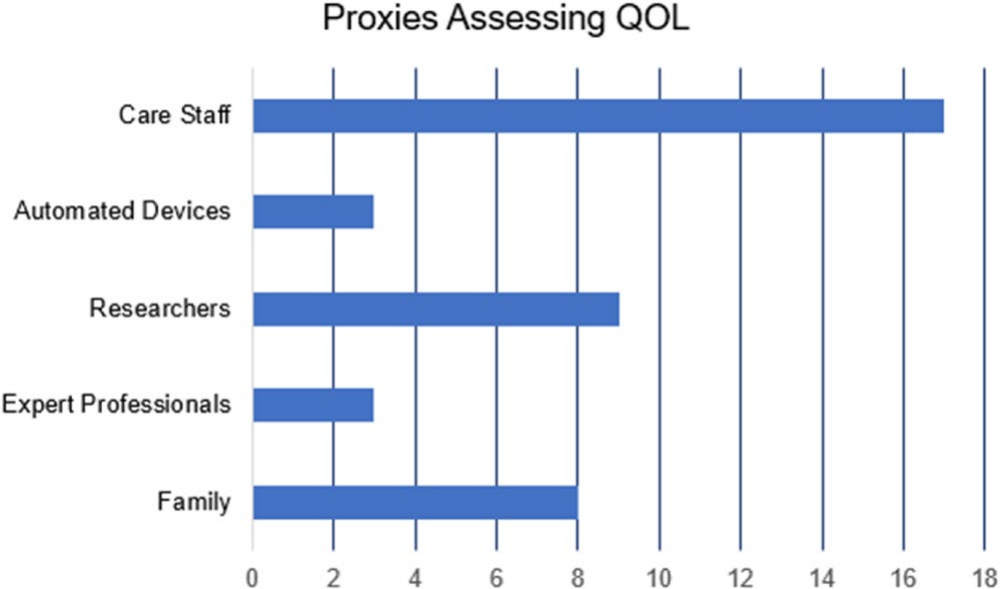
Types of Proxies Assessing QOL.

All studies used proxies to assess QOL, some alongside persons with profound intellectual and multiple disabilities, and others without including them at all, even in a passive or observational capacity. The types of proxies involved in the studies are summarised in Figure 7.

Most studies employed carer or family proxies, either directly involved in the person's care or with whom they had a close relationship. Congruence between carer/family proxy scores was variable (Bossink et al. 2017; de Geus‐Neelen et al. 2019), with some areas having greater agreement than others. The areas eliciting most disagreement concerned subjective wellbeing domains, such as ‘discomfort’ and ‘sexual fulfilment’.

Tacit knowledge (intuitive, unarticulated understanding gained through lived experience) was widely considered key to communication, being understood and ‘heard’, making needs known and met (Nieuwenhuijse, Willems, van Goudoever, and Olsman 2023; Nieuwenhuijse et al. 2022; Matérne and Holmefur 2022; Talman et al. 2021; Bradshaw et al. 2024; de Geus‐Neelen et al. 2019).

Caring personal relationships between proxies and the person with intellectual disabilities were reported to be crucial to developing tacit knowledge, enabling subjective judgements to be made on their behalf (Nieuwenhuijse, Willems, van Goudoever, and Olsman 2023; Matérne and Holmefur 2022; de Geus‐Neelen et al. 2019). However, close relationships sometimes made it harder to be objective, with parents struggling to differentiate their QOL from that of their child(ren) (Nieuwenhuijse, Willems, van Goudoever, and Olsman 2023), and the QOL of people with profound intellectual disabilities being significantly correlated with that of their family (Bertelli et al. 2011)^.^

Care staff knowledge, competence, availability and skill to provide good active support were identified as key to the wellbeing of people with profound intellectual and multiple disabilities (Matérne and Holmefur 2022; Bradshaw et al. 2024; Beadle‐Brown et al. 2016). Carers are pivotal in: establishing good communication; delivering individually tailored, high quality care; setting goals for individual support plans; advocating for service users and ensuring their needs of daily living are met (Matérne and Holmefur 2022; Herps et al. 2016). Staff awareness of power relations between themselves and service users, and sensitivity to managing these was identified as a key part of enabling inclusion and choice (Talman et al. 2021).

Carers who knew the person really well tended to give higher QOL scores than health professionals who saw the person infrequently (Carbó‐Carreté et al. 2019; Nieuwenhuijse, Willems, van Goudoever, and Olsman 2023; Bertelli et al. 2019). In studies where researchers were proxies, QOL was generally reported to be poor (Talman et al. 2021; Vos et al. 2010a; Beadle‐Brown et al. 2016).

### 4.3 Theme 3: Utility to Demonstrate Health/Wellbeing Outcomes

#### 4.3.1 Validity, Reliability and Responsiveness

Three psychometric evaluation studies were included, which rigorously assessed statistical validity and reliability of the Personal Outcomes Scale (Tatsuta et al. 2025), San Martin Scale (Verdugo et al. 2014) and the CPADULT (Zalmstra et al. 2023). However, sensitivity to change and minimum clinically important difference (the smallest change deemed to be clinically significant, MCID) were either not mentioned (Tatsuta et al. 2025; Verdugo et al. 2014) or only fleetingly discussed (Zalmstra et al. 2023). Where MCID was considered, this was calculated statistically (0.5 × Standard Deviation of mean score) and not in relation to meaningful change for the individual (Zalmstra et al. 2023).

#### 4.3.2 Quality of Life Outcomes/Thresholds

No studies evaluated QOL in relation to postural care. Only 1 study mentioned deteriorating posture and body shape as a negative influence on QOL (Bradshaw et al. 2024). 5 studies used QOL as an outcome measure following other health‐related interventions, and their results were varied. (Bossink et al. 2017). found no significant change in overall QOL following a power assisted exercise intervention, despite improvements in some domains. By contrast, both Cameranesi et al. (Cameranesi et al. 2022) and (Navas et al. 2024, 2025) found significant positive changes across all domains of QOL following community transition from institutional life. However, Cameranesi et al. found a general decline in QOL back to pre‐transition levels in their follow up study (Cameranesi et al. 2025), noting that trajectories of QOL for individuals were highly variable.

Six studies evaluated proxy scored QOL in relation to level of intellectual disabilities. Some found that QOL score was rated lower for people with more severe/profound intellectual disabilities (Carbó‐Carreté et al. 2019; Vos et al. 2010a; Baumstarck et al. 2025; Beadle‐Brown et al. 2016; Herps et al. 2016), whilst (Bertelli et al. 2019) found that people with intellectual disabilities had higher QOL than those without. (Carbó‐Carreté et al. 2019). noted that proxies who knew the person well scored QOL more highly than those who knew them remotely. Older people with profound intellectual and multiple disabilities were reported to experience worse QOL than younger people (Herps et al. 2016). None of the studies set thresholds for ‘good’ or ‘poor’ QOL, so participants scores were assessed relative to each other as ‘better’ or ‘worse’.

## 5 Discussion

### 5.1 Characterisation of QOL

QOL is inherently experienced subjectively and thus is a difficult concept to construct from outside the perspective of the person experiencing it. There is no single accepted definition or description of QOL for people with profound intellectual disabilities—definitions of QOL adopted by studies tended to be driven by the choice of outcome measure rather than the measure chosen to reflect a particular definition. This may reflect the limited choice of suitable outcome measures available, with researchers compelled to use the ‘best available fit’—even if the ‘fit’ is poor.

There is consensus in the literature that QOL is multifaceted, and comprises elements related to physical, psychological, social, and material wellbeing. Emphasis is given to social inclusion and participation, daily living activities, and interpersonal relationships. The language used to describe these elements is varied, and there is disagreement about how descriptors should be used. This reflects the inter‐relatedness of different aspects of wellbeing and highlights the difficulty of attempting to insert discrete categories into a broad concept that permeates all aspects of living.

The preference in the literature for quantitative rather than descriptive characterisation of QOL inevitably means that the meaning of QOL to individuals with profound intellectual disabilities is rarely explored. Measures based on observations of the person and their behaviour in their environment(s) do not necessarily reflect how that person is experiencing them. Further, studies rarely justify which components of QOL are assessed/included in terms of what is relevant or meaningful; rather, they tend to include what is feasible to observe or measure. Whilst this is often framed as a pragmatic decision, and the difficulty of exploring meanings for people unable to self‐report is cited, numbers without context are problematic. Attempting to quantitatively express a naturally qualitative and abstract concept will always be challenging. When a person cannot self‐report their experiences, alternative approaches are required.

### 5.2 Assessment and Measurement of QOL

It is widely accepted in the literature that using proxies is necessary when assessing QOL for people with profound intellectual disabilities, and general agreement that people who know them well are best placed to do this. The nature of the relationship between the proxies and the participants seems important—proxies in the Bertell et al. (Bertelli et al. 2019) study appraised QOL of people with intellectual disabilities that they knew well, and people without intellectual disabilities with whom they were only acquainted—possibly leading to an under‐estimation of those not so well known.

Additionally, outcomes appear more reflective of what proxies felt comfortable to comment upon, or was most practicable to observe than which areas were most important to the participants, with the least mentioned descriptors being the most subjective.

It is clear that proxies are a potential confounding factor—seven studies have attempted to compensate for this by using more than one type of proxy (Baumstarck et al. 2025; Bossink et al. 2017; Beadle‐Brown et al. 2016; Verdugo et al. 2014; Dairo et al. 2017; Zalmstra et al. 2021). Since people with profound intellectual disabilities are unable to self‐report their QOL, and therefore cannot agree or challenge these findings, there is no basis for declaring any group of proxies in the literature as more or less accurate.

Proxy reporting by automated devices, may give the most objective data, but are not available in every setting and require training to set up, calibrate, operate and interpret, which all impact cost and time resources needed. Thus, these methods may lack feasibility in everyday practice. Further, physiological data are only helpful when interpreted in the context of environmental stimuli and tacit knowledge of the individuals concerned for example, a raised heart rate may reflect pain, excitement or distress depending on the context and meanings for that person.

Rating scales are the most commonly used measures of QOL for people with profound intellectual disabilities. Scales aiming to capture total QOL are too broad to be responsive enough to detect changes brought about by interventions that influence a single area. Similarly, scales that measure a single aspect of QOL (e.g., pain, activity), may be very responsive to that domain, but not reflective of total QOL. There is no consensus on how to effectively select, utilise or combine QOL tools to measure outcomes of health interventions for people with profound intellectual disabilities. There is also a lack of clarity about whether changes (or lack of change) in QOL scores should be interpreted as improvement, maintenance, slowed or accelerated deterioration.

Scoring systems tend to assume that either a particular score threshold is required to be ‘satisfactory’ or that a change between 2 consecutive scores is a positive outcome. The initial assumption is problematic, since there were no agreed definitions or thresholds for good, poor and meaningful changes to QOL for people with profound intellectual disabilities. In the latter case, the outcome can only be said to be ‘better’ or ‘worse’ than the initial measure, not ‘good’ or ‘poor’ in and of itself.

Most studies rated the QOL of people with profound intellectual disabilities as poor. Whilst the process(es) and reasoning for reaching these conclusions are unclear, it is consistent with the health and social inequalities experienced by people with intellectual disabilities and the lower value assigned to them by society. Poor QOL was associated with poor physical and mental health, but also acknowledgement that a person with profound intellectual disabilities may maintain a good QOL, even in the presence of significant illness or disability (Nieuwenhuijse et al. 2020; Vos et al. 2010a).

Interpretation of the descriptors of QOL are subject to understanding of varied culturally and contextually derived uses of language making comparison a complex task. Even where the same words are used, meanings can be very different. As an example, take the term ‘material wellbeing’, which is used in SPOS, FQOL, San Martin Scale and QOL‐PMD measures. The San Martin Scale has 11 components relating to ‘material wellbeing’, which include aspects of physical safety of the person, environmental adaptations to meet their needs, personal space and belongings, technical aids and material goods (Verdugo et al. 2014; Gómez et al. 2015). The QOL‐PMD uses ‘material wellbeing’ to cover safety and accessibility of the environment and means of survival such as nutrition (Bossink et al. 2017; de Geus‐Neelen et al. 2019). SPOS and FQOL define it in terms of financial security (Carbó‐Carreté et al. 2019; Bertelli et al. 2011). Arguably, all are appropriate items to assess different aspects of material wellbeing, but it is possible to see that a person could be financially secure, but poorly nourished or well‐nourished but lacking physical safety. Thus, different scales rating ‘material wellbeing’ could give very different impressions of this aspect of QOL. Additionally, some terms such as ‘special attention’ and ‘optimal’ are highly subjective.

### 5.3 Use and Interpretation of QOL Measures

Bossink et al. (Bossink et al. 2017) interpreted a lack of change in QOL score to mean the intervention did not influence QOL; however, there are 2 alternative explanations—either the QOL instrument was not sensitive enough to detect change, or the intervention was instrumental in *maintaining* QOL. The community transition studies (Cameranesi et al. 2022; Navas et al. 2024) involved changes to the participants' whole way of living, so it is unsurprising that multiple areas of wellbeing would be affected. Dairo (Dairo et al. 2017) used a more specific tool to measure responses to their physical activity intervention (IPAQ). This was more sensitive to change, but less reflective of QOL as a whole. This serves to highlight the difficulties of using such a multifaceted concept as an outcome measure, due to the inherently high number of moderator variables.

MCID is based on the assumption that a measure or score should change in response to an intervention, and be unchanged without intervention, with the aim of intervention always being improvement (Kovach 2019; Abrams et al. 2006). In people who have progressive conditions and multi‐morbidity, this may not be realistic, with interventions directed more towards clinical stability and prevention or slowing of deterioration. In other words, if stability (i.e., no change in score) is the desired outcome (and therefore, clinically important), then there can be no *minimum* clinically important difference—a difference of 0, in this case, is a clinically important outcome.

These latter concepts are harder to demonstrate without comparison to data concerning the natural course/progression of the QOL of people with profound intellectual and multiple disabilities without interventions. Whilst developments such as the LeDeR project (annual report of causes of death in people with intellectual disabilities and/or autism) (NHS England 2023) have resulted in the availability of more mortality and morbidity data pertaining to profound intellectual disabilities, there is no way to know what health interventions those people received in relation to their QOL in the course of their lives. This is very different to more common conditions such as cancer, where symptom burden and health‐related quality of life is well documented across large populations, both with and without intervention (Nuffield Trust 2021).

### 5.4 Strengths and Limitations

The strengths of this review lie in the systematic approach to identifying, appraising and synthesising the data from current research literature, following an established process, the use of multiple databases and an internationally recognised QOL instrument as a basis for comparison.

One limitation is that a number of authors contributed to more than one research paper, meaning that the articles reviewed are attributable to a relatively small pool of researchers. This indicates the lack of contemporary research in this area, particularly in the UK where only 3 relevant studies have been published since 2010.

## 6 Conclusions

Establishing the effectiveness of postural care interventions to improve and maintain QOL is essential to providing evidence‐based care, achieving optimal outcomes and justifying expenditure on such provision for people with profound intellectual and multiple disabilities.

The emerging field of QOL research for people with profound intellectual disabilities is providing valuable insights into their experiences and interactions with their environments to support provision of high‐quality care. Acknowledging the breadth of the concept of QOL, researchers have been careful to include as many influencing factors as possible; however, this brings some limitations to their usage.

Current QOL scores cannot be interpreted to indicate a ‘good’, ‘acceptable’ or ‘poor’ QOL, instead providing a ‘snapshot’ assessment which must be repeated in order to establish if the situation is better, worse or the same as before. As a stand‐alone way of measuring QOL in relation to a health intervention, this is problematic for several reasons.

First, if the significance of change in score is calculated statistically, but is not related to any actual change (or lack thereof) in the person's experience, it lacks practical utility to determine meaningful improvement, stability or deterioration. Similarly, a person may perceive a large change in their QOL, but a rating scale may not be sensitive enough to reflect this.

Second, standardised ratings of QOL take no account of what type, rate and scale of change is meaningful or realistically achievable for the individual—whether the aim is improvement, stability, or slowed decline. Without this, interpretation of QOL scores is problematic.

Third, they cannot easily be used to show the benefits of a health intervention on QOL for people who by virtue of a progressive health condition or accelerated ageing process are anticipated to experience a decline in their wellbeing. The scores get worse with deterioration, but there is no way to show whether this deterioration has been slowed by the intervention.

Fourth, scores based on physical and mental health ignore the possibility that good QOL may still be achievable despite disability or poor physical and mental health.

There are currently no QOL measures that achieve the required balance between breadth of inclusion and specificity to enable responsiveness to change in relation to postural care interventions for people with profound intellectual disabilities. This represents a gap in the current knowledge base.

### 6.1 Recommendations

Evaluation of success in relation to postural care outcomes should include but not be restricted to mental and physical health outcomes, alongside QOL evaluation.

Where standardised QOL scores are used to evaluate health interventions, due consideration must be given to whether the tool is sensitive enough to reflect changes brought about by the intervention. If this is questionable, other ways of determining efficacy should be considered, which acknowledge the specific context for the individual with clear, realistic, person‐centred goals against which success can be benchmarked.

A new tool or system is needed that can assist families and carers to communicate meaningful information about the QOL of their family member/service user to those who hold decision‐making power. It is important that such tools are readily available, easy to use, unambiguous in their language and lead to shared understanding, as well as being flexible enough to reflect individual needs, values and goals.

Further research is required to explore the relationship between postural care and quality of life, and how this can be best demonstrated to support development of such tools and systems. These tools and systems can then be used to provide evidence for the clinical effectiveness of interventions and to support appropriate provision.

## Funding

The authors have nothing to report.

## Ethics Statement

Ethical approval for this research was obtained from the University of Chester research ethics committee—see enclosed letter dated 15th March 2024.

## Conflicts of Interest

The authors declare no conflicts of interest.

## Data Availability

Data sharing not applicable to this article as no datasets were generated or analysed during the current study.

## Appendix A

**TABLE A1:** Participant characteristics by study.

Study lead author/date	Participant characteristics				
	*n* =	Persons with intellectual disabilities/proxies	Age range (years)	% Male (M)	Setting/country
				% Female (F)	
Baumstarck et al. (2025)	76	All participants have profound intellectual disabilities, with 52% having severe physical disability	Mean 25.4	M 49% F 51%	Residential care (France)
Beadle‐Brown et al. (2016)	110	Severe intellectual disabilities (73%)	20–82	M 52% F 48%	Residential care (UK)
		Profound intellectual and multiple disabilities (22%)			
Bertelli et al. (2011)	27	Level of intellectual disabilities unclear	27–50	M 67% F 33%	Living with family (Italy)
Bertelli et al. (2019)	92	Severe intellectual disabilities	Mean 31.9	M 62% F 38%	Residential care (Italy)
	34	Without intellectual disabilities	Mean 28.5	M 47% F 53%	
	48	Proxies	Mean 40.2	M 45% F 55%	
Bossink et al. (2017)	37	Profound intellectual and multiple disabilities	4–60	M 67% F 33%	Community (Netherlands)
Bradshaw et al. (2024)	166	Family members/paid carers reporting on people with profound intellectual and multiple disabilities 16–69 years	not stated	Not stated	Community (UK)
Cameranesi et al. (2022)	33	Profound intellectual and multiple disabilities	23–57	M 60% F 40%	Residential care (Canada)
Cameranesi et al. (2025)	59	People with profound intellectual and multiple disabilities	13–62	M 52% F 48%	Residential Care (Canada)
			Mean 40		
Carbó‐Carreté et al. (2019)	529	Included whole spectrum intellectual disabilities	16–66	M 56% F 44%	Autonomous communities (Spain)
		(Severe/Profound intellectual and multiple disabilities 11.3%)			
Dairo et al. (2017)	20	Severe/Profound intellectual and multiple disabilities *n* = 13	20–70	M 50% F 50%	Community (UK)
de Geus‐Neelen et al. (2019)	51	Severe/profound intellectual and multiple disabilities	17–85	M 39% F 61%	Community (Netherlands)
	51	Family members	39–75	M 21% F 68% other 11%	
	51	Care staff	24–54	M 15% F 85%	
Enninga et al. (2023)	57	Profound intellectual and multiple disabilities	24–79	M 49% F 51%	Community (Netherlands)
Gómez et al. (2015)	12	Expert professionals	27–53	Not stated	Academic institution (Spain)
Herps et al. (2016)	209	Adults with intellectual disabilities 32% severe or profound intellectual disabilities	20–83	M 49% F 51%	Community (Netherlands)
Matérne and Holmefur (2022)	13	Care staff and managers	32–66	M 16% F 84%	Community (Sweden)
Navas et al. (2024)	54	People with intellectual and developmental disabilities 53.7% profound or severe intellectual disabilities	20–70	M 57.4% F 42.6%	Institution (Spain)
Navas et al. (2025)	162	People with intellectual disabilities 29% profound or severe intellectual disabilities	18–76	M 60% F40%	Institutions (Spain)
Neveu et al. (2025)	567	French speaking participants with intellectual disabilities 173 mild, 207 moderate, 187 severe to profound	Mean 46	M 67% F 33%	Residential care (Belgium)
Nieuwenhuijse et al. (2020)	7	Physicians	Mean age 50	Not stated	Hospital (Netherlands)
Nieuwenhuijse et al. (2022)	11	Carers	25–50	F 100%	Various care settings (Netherlands)
Nieuwenhuijse, Willems, and Kruithof (2023)	22	Parents	Not stated	M 54% F 46%	Children aged 9–51 years
Talman et al. (2021)	4	Profound intellectual and multiple disabilities	20–49	M 75% F 25%	Group home (Sweden)
Talman et al. (2019)	27	Care staff	22–65	Not stated	Community (Sweden)
Tatsuta et al. (2025)	174	102 people with intellectual disabilities (10% severe) and 72 support workers	18–50+	M 58% F 42%	Community (Denmark)
Verdugo et al. (2014)	1770	Profound intellectual and multiple disabilities	16–77	M 56% F 44%	Community (Spain)
	399	Professional carers, family members	Not stated	Not stated	
Vos et al. (2010a)	360	Severe/Profound intellectual and multiple disabilities	Over 18	Not stated	Community (Belgium)
	354	Proxies			
Vos et al. (2010b)	3	Profound intellectual and multiple disabilities	23–52	M 66% F 34%	Community (Belgium)
Vos et al. (2013a)	27	Severe/Profound intellectual and multiple disabilities	22–54	M 49% F 51%	Community (Belgium)
Vos et al. (2013b)	27	Severe/Profound intellectual and multiple disabilities	22–54	M 49% F 51%	Community (Belgium)
Zalmstra et al. 2021)	26	Support workers, Parents, Professional experts	Not stated	M 13% F 81% not stated 6%	Community (Netherlands)
Zalmstra et al. (2023)	47	Caregivers	19–23	M 23% F 77%	Community (Netherlands)

**TABLE A2:** Characteristics of included studies.

Author/year/reference/location	Aims/research questions	Theoretical/Conceptual framework/design/methodology	Relevant findings	Strengths/limitations/appraisal score
(Baumstarck et al. 2025) Quality of life among individuals with profound intellectual and multiple disabilities: crossed perspectives of institutional caregivers and parents (France)	Comparison between assessments of QOL by parents and institutional caregivers of people with profound intellectual disabilities	Post positivist Quantitative descriptive cohort study reporting on French cohort of a larger multinational study. PolyQoL questionnaire GMFCS Functional Independence Measure Brunet Lezine scale (Neurodevelopmental status) Number of devices Number of medications	Both groups rated health QOL higher than social QOL. Institutional care givers generally rated global and social QOL lower than parents, particularly associated with increasing age, severity of disability, and being in residential care.	All participants have profound intellectual disabilities. Age range not reported, except participants aged over 3 years. Impossible to tell how many adults vs. children participated Young age of participants (mean age 25 years) does not account for QOL factors associated with ageing, or those which might vary between adults and children. MMAT score 6/10
(Beadle‐Brown et al. 2016) Quality of Life and Quality of Support for People with Severe Intellectual Disability and Complex Needs (UK)	‐ Describe quality of life for people with severe intellectual disabilities—Describe quality of support received by these people ‐Describe predictors of good outcomes	Post positivist Mixed methods Cross‐sectional study Using qualitative data in a quantitative way, Categorising behaviours/activities rather than exploring meanings. Managers questionnaire SABS, QSI, ABC, CSRI, EMARO	40% of participant's time engaged in meaningful activities, 25% receiving contact from staff. 6% receiving support to be engaged. 34% received consistently good active support from staff. QOL and quality of support generally poor People with more severe intellectual disabilities experienced poorer outcomes, spent less time in contact with other people and less time being meaningfully engaged. Good active support best predictor of good QOL	No definition QOL QOL measured by percentage of time engaged in meaningful activity – not described how they determined what was meaningful to the participants. Very heterogeneous group. Assumption that level of contact time is positively correlated with good outcomes that is, That contact is a good thing in itself. No consideration of need for rest/low stimulation time All measures taken by researchers, staff, managers not people with intellectual disabilities. MMAT Score 7/10
(Bertelli et al. 2011) Relationship between individual quality of life and family quality of life for people with intellectual disability living in Italy. (Italy)	To describe the relationship between quality of life of individuals with intellectual disabilities and members of their families	Post positivism—Quantitative Cross‐sectional study FQOLS Survey and QOLIP Scores compared for similarities/differences/correlations.	Family ratings of QOL low in ‘support from others’ ‘community interaction’ Rated higher ‘family relationships and family health’. Individual QOL rated lowest in ‘spiritual being’ and highest in ‘physical being’. Statistically significant correlations between some individual and family domains—financial well‐being, family relationships, support from service and support from others	QOL examined in general terms, not in terms of health related QOL. QOL defined in terms of outcome tools used. Level of intellectual disabilities not disclosed, except to say that some participants were unable to articulate their own ideas, so the tool was completed by 2 or more proxies. Different domains assessed for individuals and families. Domains assessed include Being, Belonging, and Becoming for the individual. MMAT score 5/10
(Bertelli et al. 2019) Impact of severe intellectual disability on proxy instrumental assessment of quality of life (Italy)	Investigate the impact that presence of severe intellectual disabilities has on proxy attribution of QOL	Post positivism—Quantitative non‐randomised trial QOLIP administered to group with intellectual disabilities and control group without	QOL rated significantly higher for people with intellectual disabilities than general population in every domain except psychological being. Decision making was significantly lower for the group with intellectual disabilities. Opportunities rated higher in general population group in spiritual and social belonging. Well informed proxies attribute high QOL scores—low prejudice or artificially high for other reasons?	Relatively large sample of participants with severe ID (*n* = 92) Relatively small sample without intellectual disabilities (*n* = 48)—unevenly matched and unlikely to be representative of population. Domains assessed similar in both groups. People with intellectual disabilities were well known, those without were only acquaintances. MMAT score 5/10
(Bossink et al. 2017) A power‐assisted exercise intervention in people with profound intellectual and multiple disabilities living in a residential facility: a pilot randomised controlled trial. (Netherlands)	‐Assess feasibility of full RCT ‐Evaluate efficacy of power assisted exercise intervention for people with profound intellectual disabilities	Post positivist Pilot Randomised Controlled Trial Some outcomes single blinded, others not blinded. Intervention group and control group Observational measures including: Functional abilities (BAS), alertness (Alertness Observation List), Body Mass Index, muscle tone (Modified Ashworth Scale), oxygen saturation and cardiovascular fitness (pulse oximetry) and QOL (QOL‐PMD)	Trial design feasible and acceptable to participants Improved oxygen saturation in intervention group No difference in other measures including QOL	Small sample size but appropriate for pilot. Heterogenous group despite attempts at reducing this. Lack of wider evidence base about normal responses to exercise in profound intellectual disabilities, makes interpretation of results difficult. Findings contradict those of other studies about effects of powered exercise. QOL based on aggregate of observational measures—and considered as separate to physical/physiological measures. MMAT score 6/10
(Bradshaw et al. 2024) What would have helped people with profound intellectual and multiple disabilities in the UK during COVID‐19? (UK)	Explore factors that make life difficult and make life better for people who have profound intellectual and multiple disabilities, in relation to the COVID‐19 pandemic	Pragmatism Qualitative descriptive Reporting on the 2nd cohort of a larger study Online survey with a mixture of closed questions, Likert scale response questions, and open questions	Factors which negatively affect QOL: Social isolation, loss of routine, mental illness, deteriorating physical health, loss of life skills, boredom, deterioration in body shape. What would have helped: Consistency, opportunities for social contact, meaningful activities, better access to health and social care services particularly physiotherapy, hydrotherapy and rebound therapy, staff training and staff team working	Relatively large sample size *n* = 166 (all proxies) broadly representative of carers both paid and unpaid. No specific validated tool or method to assess QOL of the individuals involved. Nevertheless a pragmatic exploration of the opinions of families and carers about factors which negatively and positively influence QOL of people with profound intellectual and multiple disabilities. Values input of experts by experience, but excludes views of professionals. Specific to the COVID 19 pandemic, but applications to life outside this and comparisons to the general population explored. MMAT score: 6/10
(Cameranesi et al. 2022) Changes in the quality of life of persons with profound intellectual and multiple disabilities following community transition: A Canadian study. (Canada)	Measure changes in QOL of adults with profound intellectual disabilities following transition from a large institution to smaller community homes	Post positivism Quasi‐experimental, longitudinal, quantitative non‐ randomised design Proxy assessed (scored) QOL in eight domains of San Martin Scale pre transition and > 6 months post transition	Significant improvement across all 8 domains of QOL post transition: Self determination Emotional wellbeing Physical wellbeing Material wellbeing Rights Personal development Social inclusion Interpersonal relations	Sample size small (Matérne and Holmefur 2022) but appears representative. Validated tool for use with people who have intellectual disabilities. Confounding variables discussed and addressed. MMAT score 8/10
(Cameranesi et al. 2025) Quality of life trajectories in persons with intellectual disabilities undergoing community transition in Central Canada: A longitudinal repeated measures study (Canada)	Track changes in QOL over time of adults with profound intellectual disabilities following transition out of institutions to community homes	Post positivism Repeated measures quasi‐experimental time series study Proxy completed San Martin Scale pre transition and post transition at 6–48 months, then at yearly intervals for 2 years.	Significant changes across all 8 domains between time points with highest scores immediately post transition, followed by a general decline to pre‐transition levels by the final assessment. Individual trajectories variable. Some sustained gains in QOL whilst others did not.	Moderate sample size (Cameranesi et al. 2025) Use of validated tool Proxies were family or support staff who knew the person well Some pre transition scores completed retrospectively potentially introducing recall bias Variability of time scale for first post transition scoring may have affected how the person's experience of transition was reflected. MMAT Score 8/10
(Carbó‐Carreté et al. 2019) Impact of the intellectual disability severity in the Spanish Personal Outcomes Scale (Spain)	To examine impact of severity of intellectual disabilities on attributed QOL	Quantitative validation study Spanish Personal Outcomes Scale comparing scores as assessed by family, professionals and self‐reported	Professionals tend to estimate QOL as being lower in individuals with more severe intellectual disabilities, particularly with regard to rights, personal development and self‐determination.	Only 11.3% of participants had severe or profound intellectual disabilities. Those with most severe intellectual disabilities cannot express their opinion to agree/challenge attributed QOL. This group need further study. MMAT score 6/10
(Dairo et al. 2017) A feasibility study into the measurement of physical activity levels of adults with intellectual disabilities using accelerometers and the International Physical Activity Questionnaire (UK)	To explore the feasibility of ‐Recruiting people with intellectual disabilities to take part in research—the characteristics of those who take part—congruence between different measures of physical activity and self/proxy reports of physical activity	Pragmatist Mixed methods Cross sectional feasibility study Qualitative data—self reported, and proxy reported accounts of physical activity using IPAQ. Quantitative data using accelerometer	Accelerometers agree with IPAQ in identifying those who were active. IPAQ seems to underestimate activity levels but was more acceptable to participants. There was strong agreement between self‐reported and proxy reported IPAQ. IPAQ's can be used to evaluate physical activity levels for health related QOL.	Assessed small number of people (*n* = 20), of whom slightly more than half had severe or profound intellectual disabilities. Measures only one aspect of health related QOL. Measures validated for the population and seemed acceptable to participants, with high level of congruence. MMAT score 7/10
(de Geus‐Neelen et al. 2019) Perceptions of staff and family of the quality of life of people with severe to profound intellectual disability (Netherlands)	Explore level of agreement between family and staff acting as proxies for determining quality of life for people with severe to profound intellectual disabilities	Post positivist Quantitative cross‐sectional design Compared ratings of family and staff proxies using QOL‐PMD tool (Likert scale ‐agree/partly agree/disagree/don't know/not applicable) Physical wellbeing (19 items) Social wellbeing (21 items) Communication and influence 16 items) Development15 items) Activities (15 items) Similarity assessed with inferential statistics and Wilcoxon signed rank rest	Proxies agree in some areas but not others. They differ most widely on area of subjective wellbeing. Proxy assessment may not be reliable indicator of wellbeing. Some areas of wellbeing rated as ‘not applicable to the client’ including pain, discomfort, sexual fulfilment	Small sample *n* = 51 from a single health organisation. Data where there was disagreement about applicability of items was not included or explored. No capacity for narrative discussion of QOL by participants to capture elements the QOL‐PMD may have missed. Causes of differences not explored or explained. No indication of which part of the dyad was more reliable. MMAT score 7/10
(Enninga et al. 2023) Reliable assessment of pain behaviour in adults with profound intellectual and multiple disabilities: The development of an instruction protocol (Netherlands)	Develop and test an observational tool for detection of pain in people with profound intellectual disabilities	Pragmatic approach Quantitative cross‐sectional design Calibration of pain behaviours assessment tool and assessment of inter‐rater reliability *N* = 57 adults with profound intellectual disabilities age 24–79 years	Behaviours which indicated pain include: Tense face, eyes squeezed, raising upper lip, grimace, fearful look, non‐functional movement, vocalisations, changes in facial colour, movements of lips/tongue, change in movements, stiffness/spasticity. Better recognition of pain improves quality of life	High level of calibration needed means scoring on a day‐to‐day level may be impractical. Assessment via video—perhaps less sensitive than face to face—but also allows for repeat viewing, which cannot be done in real life setting. QOL not specifically assessed—focus on single component of pain only. Claims that QOL will be improved using this tool not supported. MMAT score 6/10
(Gómez et al. 2015) Operationalisation of quality of life for adults with severe disabilities (Spain)	Aim for expert consensus on valid indicators of quality of life for adults with severe disabilities	Pragmatism Mixed methods Grounded theory Modified Delphi study Schalock and Verdugo's eight domains used as a framework. Self‐determination, emotional wellbeing, physical wellbeing, material wellbeing, personal development, rights, social inclusion, interpersonal relationships	Created a pool of 118 items across 8 domains to be included in a new instrument for measuring quality of life of persons with severe disabilities.	Encompasses all severe disabilities, not limited to intellectual disabilities but draws heavily on intellectual disability research and literature. Doesn't account for views of people with intellectual disabilities or their families/carers. Not all domains appropriate for all individuals Tool potentially lengthy/time consuming No guidance about who (which proxies) complete the tool. MMAT score 8/10
(Herps et al. 2016) Individual support plans of people with intellectual disabilities in residential services: content analysis of goals and resources in relation to client characteristics (Netherlands)	Establish differences in care support goal planning in relation to QOL dependent on severity of intellectual disabilities	Pragmatism Mixed methods Qualitative and quantitative data Coding used for content analysis alongside narrative analysis. Schalock and Verdugo's framework used as reference. Inter‐rater agreement assessed statistically	Older people and people with more severe intellectual disabilities had fewer goals. Of the goals they had, significantly fewer directed towards social participation, and significantly more directed at physical wellbeing	QOL of individuals not actually assessed in this study, only aspirational goals determined by proxies. Relationship between goals and actual QOL not investigated. Raises appropriate questions about the suitability of the approach to determining effectiveness of care. MMAT score 7/10
(Matérne and Holmefur 2022) Residential care staff are the key to quality of health care for adults with profound intellectual and multiple disabilities in Sweden. (Sweden)	To explore experiences of managers and staff of residential properties regarding health care services for people with profound intellectual disabilities	Interpretive Phenomenology Semi‐structured interviews design Qualitative content analysis QOL assessed in terms of access to/quality of care. Accessibility, treatment, cooperation, communication, support and participation	Quality of healthcare is dependent on residential care staff. Individually tailored support created collaboratively promotes quality of care but is hard to achieve. Participation is enabled by advocacy. Healthcare access is limited by staffing and prioritisation. Disabilities competence among staff promotes quality and safety. Communication is key to quality of care	Quality of healthcare impacts on QOL and this is the focus of this study, though other impacts are acknowledged. Focus is on staff perceptions, not people with intellectual disabilities. Sample size is small and geographically limited. MMAT score 8/10
(Navas et al. 2024) Improving Quality of Life and Reducing Behavioural Problems of People with Intellectual and Developmental Disabilities Through Deinstitutionalization (Spain)	Exploring changes in functioning and QOL for adults with intellectual disability and extensive support needs, in relation to deinstitutionalisation.	Post positivism Longitudinal, quantitative descriptive study QOL assessed by proxies using Resident Choice Scale San Martin Scale Active Support Participation Measure Prior to transition, and again at 6 months and 12 months post transition	Significant improvements in QOL as shown by increased decision making, participation and independence in daily activities, and a reduction in frequency and intensity of behavioural problems. No change in choices related to support professionals, staffing issues or meals. Significant changes is choices about household appearance/possessions, personal appearance, leisure and relationships. Increase in autonomy related to meal preparation and household chores.	Relatively small sample size (54 people aged 20–70), limiting analysis of environmental and personal variables. Only 53.7% of sample had severe or profound intellectual disability—difficult to know if/whether the results differed by level of intellectual disability. Almost 30% were not living in institutions but family homes prior to transition to community home—whether this equates to ‘deinstitutionalization’ is questionable. MMAT score 8/10
(Navas et al. 2025) Empowering lives: How deinstitutionalization and community living improve the quality of life of individuals with intellectual and developmental disabilities (Spain)	Exploring mechanisms of change and improvement in quality of life after deinstitutionalisation	Post positivism Longitudinal, quantitative descriptive study Active Support Participation Measure Resident Choice Scale San Martin Scale Prior to transition and at 9 months post transition	Significant improvements in mean scores across all variables for those who moved, compared with those who did not. This, despite ‘movers’ having higher pervasive support needs Positive changes in QOL require constant availability of opportunities to support decision making, participation and autonomy in daily activities	162 participants of whom 90 were ‘movers’ and 72 were ‘non‐movers’ making it difficult to achieve participant matching. Data collected by interviewing direct support professionals, some of whom had only known the individuals concerned for 3 months. MMAT score 7/10
(Neveu et al. 2025) Math for life: Understanding the contribution of numeracy to the quality of life of adults with mild to profound intellectual disabilities (Belgium)	To investigate whether numeracy can be considered a predictor of QOL in adults with intellectual disabilities	Post positivism Quantitative descriptive cohort study, using a subset of data extracted from a larger study. Personal Outcomes Scale (French version) Numeracy and Literacy scales derived from Vineland Adaptive Behaviour Scale II protocol	Correlations were found between adaptive behaviour and QOL, and literacy and QOL in people with severe or profound intellectual disabilities. Numeracy scores for people with severe‐profound intellectual disabilities were close to zero. Numeracy was not associated with wellbeing, but was correlated with independence and social participation at all levels of intellectual disability.	A large‐scale study (*n* = 567) including people with all levels of intellectual disabilities. Including 33% with severe or profound intellectual disabilities. MMAT score 8/10
(Nieuwenhuijse et al. 2020) Physicians' perceptions on Quality of Life of persons with profound intellectual and multiple disabilities: A qualitative study (Netherlands)	Explore perceptions of physicians on QOL of people with profound intellectual and multiple disabilities	Interpretive Phenomenology Qualitative design Purposive sampling Semi‐structured interviews Thematic analysis	Emotional elements such as contentment used to describe good QOL. Emphasis on balance between positive and negative influences on QOL Elements such as ill health, poor prognosis, or poor interpersonal relationships used to describe poor QOL	7 physicians interviewed, the majority being paediatricians or paediatric neurologists, with only a few specialists in adult intellectual disabilities. Appropriate to methodology, but limited range of views. Highlights the ethical dilemmas that result from appraisal of QOL when considering provision or withholding of interventions. MMAT score 9/10
(Nieuwenhuijse et al. 2022) The perspectives of professional caregivers on quality of life of persons with profound intellectual and multiple disabilities: a qualitative study (Netherlands)	Explore perceptions of professional carers on QOL of people with profound intellectual and multiple disabilities	Interpretive Phenomenology Qualitative design Purposive sampling Semi‐structured interviews Thematic analysis	Good QOL described in terms of happiness, pleasure, enjoyment and control. Poor QOL described in physical terms such as illness, pain, shortness of breath. QOL assessed intuitively, using tacit knowledge.	Small sample appropriate to methodology but may not be representative. Participants acknowledge the difficulty in making subjective judgements on behalf of others. Highlights the feelings of powerlessness experienced by carers when they perceive they are no longer able to provide a good QOL. Highlights the importance of relationships to gain understanding and tacit knowledge. MMAT score 9/10
(Nieuwenhuijse, Willems, van Goudoever, and Olsman 2023) Parent perspectives on the assessment of quality of life of their children with profound intellectual and multiple disabilities in the Netherlands (Netherlands)	To explore QOL of people with profound intellectual and multiple disabilities as perceived by their parents	Interpretive Phenomenology Qualitative design Purposive sampling Semi‐structured interviews Thematic analysis	Parents describe trusting, long term relationships as key to QOL. Parents perceive themselves as the best proxy to determine QOL followed by siblings. Physicians not perceived to know the person well enough to determine QOL. Health professionals only see glimpses through unusual moments (usually when person is ill)—they rarely experience ‘ordinary life’	Small sample appropriate to methodology—may not be representative. 12 of 16 ‘children’ were adult offspring. Explores the qualities required of the QOL assessor. Highlights the ethical dilemmas associated with physician assessed QOL being used for giving/withholding interventions. MMAT score 9/10
(Talman et al. 2021) Participation in daily life for adults with profound intellectual (and multiple) disabilities: How high do they climb on Shier's ladder of participation? (Sweden)	Explore how often adults with profound intellectual and multiple disabilities are able to participate in daily life with respect to being listened to and expressing views and involvement in making decisions	Descriptive Phenomenology Qualitative, observational deductive design Purposive sampling Digital video footage observing interaction between carers and service users. Observations compared to Schier's ladder of participation	Adults in this study did not reach level 5 (shared power in making decisions), and only once achieved level 4 (involved in decision making process). Limitations were felt to be due to lack of staffing, lack of understanding by staff about participants capabilities.	Very small sample *n* = 4 in a single location/context—limited representation. QOL assessed in this context in relation to participation only. Levels of participation required to achieve adequate QOL not identified. Other facets of QOL which may influence participation not discussed. MMAT score 5/10
(Talman et al. 2019) Staff members and managers' views of the conditions for the participation of adults with profound intellectual and multiple disabilities (Sweden)	Describe what participation means to adults with profound intellectual and multiple disabilities, as perceived by staff members	Interpretive Phenomenology Qualitative Purposive sampling Semi‐structured interview design Thematic analysis	What participation means to people with profound intellectual and multiple disabilities not described. Instead, conditions that influenced participation were categorised as follows—Degree of impairment, Staff knowledge about disabilities and about the individual, communication skills, time available, power, sensitivity and awareness, organisational factors	No definition of QOL or participation Research question not answered—different interview questions/technique may have helped collect more relevant data. However, data accurately reported and analysed, and non‐attainment of answer addressed. MMAT score 6/10
(Tatsuta et al. 2025) Personal Outcome Scale for persons with intellectual disability in Denmark: A preliminary study on reliability and validity	To explore psychometric properties of the Personal Outcomes Scale in the Danish context	Validity & Reliability study Purposively sampled people with intellectual disabilities and their support workers Evaluated inter‐rater reliability and internal consistency	Significant differences found in group validity analysis across disability levels and age groups. Cultural adaptations required and further validation is needed. Lack of alternative scales in Danish noted.	Minimum clinically important difference not discussed Cultural nuances identified linguistically during translation, rather than contextually or by consulting with experts in Danish culture. MMAT score 5/10
(Verdugo et al. 2014) Measuring quality of life in people with intellectual and multiple disabilities: validation of the San Martín scale (Spain)	Validation of the San Martin Scale to measure QOL in people with profound intellectual and multiple disabilities	Post positivist Quantitative Validation study Construct validity and reliability assessed statistically	San Martin Scale is a 95 item Likert scale completed by a proxy respondent. Divided into 8 domains of QOL—which were found to be reliable and valid, providing best fit to the data above unidimensional and hierarchical alternatives. 8 domains—self‐determination, emotional wellbeing, physical wellbeing, material wellbeing, personal development, rights, social inclusion, interpersonal relationships	Large sample size, very representative, wide age range, 62% of participants had severe or profound intellectual disabilities Includes aspects of QOL not included in WHO definition. QOL determined by the factors in the assessment tool. Rigorous assessment of construct validity and reliability. All assessment via proxy—views of people with intellectual disabilities not included. MMAT score 9/10
(Vos et al. 2010a) What makes them feel like they do? Investigating the subjective well‐being in people with severe and profound disabilities (Belgium)	To explore the subjective wellbeing of people with severe or profound intellectual disabilities and the personal and service characteristics that contribute to this	Post positivist Quantitative descriptive cross‐sectional design Mood Interest and Pleasure Questionnaire scores (completed by proxy informant) Regression analysis and correlation analysis	People with profound and severe intellectual disabilities had lower subjective wellbeing scores than people with mild/moderate intellectual disabilities in a previous study. Profound intellectual disabilities associated with worse subjective wellbeing than severe intellectual disabilities. Lower wellbeing scores were associated with higher incidence of medical problems, medication, constipation, feeding difficulties and level of physical support for activities of daily living	Relatively large sample across multiple organisations—limited to residential institutions, nobody who was living in the family home was included. Completion of MIPQ highly subjective on part of the proxy, and to attempt statistical analysis of scoring of something potentially unreliable is questionable. Snapshot only—no follow up. QOL is not a static phenomenon and results may have been very different if collected over time. No investigation of how proxies determined their answers. MMAT score 6/10
(Vos et al. 2010b) Do You Know What I Feel? A First Step Towards a Physiological Measure of the Subjective Well‐Being of Persons with Profound Intellectual and Multiple Disabilities (Belgium)	Attempt to correlate physiological measurements with observed emotional states in people with profound intellectual and multiple disabilities	Post positivism Quantitative Observational case series Measured skin conductance, respiration rate, heart rate, respiratory sinus arrhythmia (RSA), observed behaviour in response to staff selected positive and negative stimuli.	Participants had higher skin conductance, faster, shallower respiration and less RSA when experiencing positive emotions than negative emotions.	Underlying assumptions that particular stimuli will result in direct emotional response, and that certain emotions are positive or negative in and of themselves. Very small sample *n* = 3, so generalisability questionable—though the authors acknowledge this is a ‘first step’. Physiological measures as a proposed tool for emotional state will need to account for other causes in changes in physiological markers—about which little is currently known in this population. Not a practical tool for use on a day‐to‐day basis. MMAT score—5/10
(Vos et al. 2013a) Investigating the relationship between observed mood and emotions in people with severe and profound intellectual disabilities (Belgium)	Explore relationship between observed mood and observed emotion in people with Profound intellectual disabilities	Post positivist Hypothetico‐deductive Quasi‐experimental design MIPQ completed by proxy compared with video observation of responses to staff selected positive and negative stimuli	It is possible to distinguish mood and emotion by observation. Observed low mood associated with lower positive emotional responses. Positive relationship between total MIPQ score and emotion scores during positive stimuli, but not with negative stimuli. No relationship between mood and intensity of emotion.	Moderate sample size, non‐randomised. Individually selected positive and negative stimuli, and individual expressions of positive/negative emotion—how this was determined not discussed. MIPQ and video completed separately, so reflective of different points in time. Not clear how assessment of mood and emotion is reflective of QOL, and external factors affecting QOL not accounted for (e.g., physical health). MMAT score 6/10
(Vos et al. 2013b) See me, feel me. Using physiology to validate behavioural observations of emotions of people with severe or profound intellectual disability. (Belgium)	To determine if there is a physiological relationship between observed behaviour and emotional response	Post positivism Hypothetico‐deductive Quasi‐experimental design Physiological measurements of respiration, heart rate and movement compared with observational analysis of behaviour expressing positive or negative emotion	Similar physiological responses to positive and negative emotions as seen in general population studies. Supports the notion that affective behaviour is an indicator of emotion in people with profound intellectual disabilities.	Small sample size with limited generalisability Confounding variables such as heterogeneity of participants, excess movement affecting monitoring, differing response to same stimuli are discussed. Practicality of real‐life application not discussed. Addresses only emotional wellbeing and not other QOL factors. MMAT score 5/10
Zalmstra et al. 2021) Development and sensibility assessment of a health‐related quality of life instrument for adults with severe disabilities who are non‐ambulatory. (Netherlands)	To develop a health related QOL instrument for non‐ambulatory adults with severe motor and intellectual disabilities	Post positivist Mixed Methods design Qualitative phase informing quantitative phase. Interviews for member checking. Focus groups, e‐survey and interviews with parents and direct support professionals. Resultant tool CPADULT	Agreed facets of health related QOL—Activities of daily life/personal care (9 items) Positioning, transferring and mobility (8 items) Comfort and emotions (10 items) Communication and social interaction (10 items) Health (4 items) Overall quality of life (1 item)	Small sample size, homogenous group—perhaps limits scope for differing views, and gives false impression of early data saturation. Instrument not yet psychometrically tested. Includes both observational and subjective facets of QOL with high levels of agreement, robustly member checked and explained. MMAT score 9/10
(Zalmstra et al. 2023) Assessing the reliability and validity of a health‐related quality of life instrument, CPADULT, in a Dutch sample of adults with severe disabilities who are non‐ambulatory. (Netherlands)	Assessing validity and reliability of CPADULT tool for people with severe disabilities who are non‐ambulatory	Post positivism Validation study Test/retest reliability, measurement error, content and construct validity	CPADULT has sufficient reliability and validity as a proxy measure of health related QOL for adults with severe disabilities who are non‐ambulatory	Potential to be applied in both everyday settings and in research settings, both individually and at group level. Potential confounding factors addressed. Inclusion of domains with lower validity explained and robustly justified. MMAT score 9/10

**TABLE A3:** Codes grouped by theme.

Codes—descriptors	Codes—methods and tools	Codes—outcome measurement
Comfort Pain Respiratory function Epilepsy/seizure activity Physical health/being/wellbeing. Safe swallow Physical activity Cardiovascular function Muscle tone Excessive sleeping Happiness/enjoyment/pleasure Mood Emotional state/being/wellbeing. Being understood Alertness Degree of impairment Body composition Positioning/transfers/mobility Functional ability Participation in activities of daily living Personal care Engage in meaningful activity. Cooperation Self‐determination/control/making choices. Power relations Communication/influence/being listened to Interpersonal relationships Trust Social interaction/inclusion belonging. Support available. Rights Sexual fulfilment Material wellbeing Accessibility of care & treatment Care organisation quality Staff/carer availability Proxy knowledge/competence Personal development/growth Values Body shape Numeracy Literacy	CPADULT Respiration rate Heart rate Movement MPIQ Skin conductance Observed behaviour, Observation, Categorization of types of interaction San Martin Scale Subjective description Tacit Knowledge Number and quality of goals set in care planning. Presence/absence pain behaviours QOL‐PMD IPAQ SPOS/FPOS/DPOS BAS AOL BMI MAS Pulse oximetry QOLIP FQOLS Time engaged in activity Resident Choice Scale Active Support Participation measure POLYQoL GMFCS FIM Brunet‐Lezine Scale VABS11	Validity Reliability Responsiveness Sensitivity Feasibility Acceptability

**TABLE A4:** WHOQOL domains and facets (World Health Organisation 2012).

WHOQOL domain		WHOQOL facets	
1	Physical capacity	1a	Pain and discomfort
		1b	Energy and fatigue
		1c	Sleep and rest
2	Psychological	2a	Positive feelings
		2b	Thinking, learning, memory and concentration
		2c	Self‐esteem
		2d	Bodily image and appearance
		2e	Negative feelings
3	Level of independence	3a	Mobility
		3b	Activities of daily living
		3c	Dependence on medications or treatments
		3d	Work capacity
4	Social Relationships	4a	Personal relationships
		4b	Social support
		4c	Sexual activity
5	Environment	5a	Physical safety and security
		5b	Home environment
		5c	Financial resources
		5d	Health & social care accessibility and quality
		5e	Opportunities for acquiring new information and skills
		5f	Participation in and opportunities for recreation/leisure activities
		5 g	Physical environment (pollution/noise/traffic/climate)
		5 h	Transport
6	Spiritual/religion/personal beliefs		No facets

**TABLE A5:** Abbreviations of rating scales and tools.

Abbreviation	Meaning
ABC	Aberrant behaviour checklist—a behaviour rating scale for the assessment of treatment effects (Aman et al. 1985)
AOL	Alertness observation list (Munde et al. 2011)
CSRI	Client service receipt inventory—a comprehensive assessment of all services received by an individual (DIRUM 1997)
EMARO	Engagement in meaningful activity and relationships survey (Mansell and Beadle‐Brown 2005)
FIM	Functional Independence Measure (Baumstarck et al. 2025)
FQOLS	Family Quality of Life Survey 2006 (Brown et al. 2006)
GMFCS	Gross Motor Function Classification Scale—rates the physical abilities of a person with cerebral palsy (Palisano 1997)
IPAQ	International Physical Activity Questionnaire (IPAQ 2002)
MIPQ	Mood, Interest and Pleasure Questionnaire (Ross and Oliver 2003)
QOLIP	Quality of Life Instrument Package (Brown et al. 1998)
QOL‐PMD	Quality of Life‐Profound Multiple Disability (Petry et al. 2009)
QSI	Quality of Social Impairment question from the handicaps behaviours and skills schedule (Wing and Gould 1978)
RCS	Resident Choice Scale (Hatton et al. 2004)
SABS	Short form of adaptive behaviour scale (Hatton et al. 2001)
San Martin	San Martin Scale (Verdugo et al. 2013)
Schiers Ladder	Schiers ladder of participation (Talman et al. 2021)
POS	Personal Outcomes Scale (Carbó‐Carreté et al. 2019) SPOS Spanish, FPSOS French, DPOS Danish
VABS11	Vineyard Adaptive Behaviour Scale (Neveu et al. 2025)
WHOQOL	World Health Organisation Quality of life tool (World Health Organisation 2012)

**TABLE A7:** Rating scales mapped into WHOQOL domains and facets.

WHOQOL domains and facets	Rating scales
1. Physical capacity	
1a. Pain and discomfort	FQOL—health PMD‐QOL—physical health status is good, no discomfort from medications or feeding problems, no pain San Martin Scale—pain controlled, SPOS—Physical wellbeing POLYQoL—Physical wellbeing
1b. Energy and Fatigue	QOL‐PMD—optimal use physical abilities
1c. Sleep and Rest	IPAQ—question 7—time spent sitting QOLIP—Sleep QOL‐PMD—well rested in the morning, never asleep during the day due to lack of stimulation
2. Psychological	
2a. Positive feelings	QOLIP—psychological health and feelings QOL‐PMD—uses socio‐emotional abilities, expresses affection, San Martin Scale—lists of known behaviours that indicate emotional expression SPOS—emotional wellbeing MIPQ—mood, pleasure, interest POLYQoL ‐psychological wellbeing
2b. Thinking, learning, memory and concentration	ABS—level of ID QOL‐PMD—optimal use of intellectual abilities AOL—alertness Brunet Lezine Scale—neurodevelopmental status
2c. Self‐esteem	QOLIP—‘being’ self‐concept. Self esteem San Martin—advice and support regarding sexuality
2d. Bodily image and appearance	QOLIP ‘being’—appearance, grooming, self‐consciousness
2e. Negative feelings	QOLIP—‘being’ psychological health and feelings MIPQ—mood, pleasure, interest
3. Level of independence	
3a. Mobility	IPAQ—questions 5&6—time spent walking QOLIP—fitness & mobility QOL‐PMD—optimal use of physical abilities, preferences of mobility and posture VABS II—Gross & fine motor skills GMFCS—Gross motor skills, ambulation & mobility Brunet Lezine Scale—neurodevelopmental status
3b. Activities of daily living	EMARO—% time spent on simple, household and self‐care activities, participation and choice making QOLIP—‘being’ hygiene, nutrition QOLIP—‘Becoming’—regular domestic, work and health activities QOL‐PMD—choices in activities and actions, opportunity for independent actions, express preferences for nourishment, actively engaged in group and individual activities, not limited by sensory issues, San Martin Scale—choices enabled and respected, activity program that reflects preferences SPOS—independence, self determination Schiers Ladder—involvement, shared power/responsibility VABS II—gross and fine motor skills, personal and domestic life FIM—Functional independence/assistance needed
3c. Dependence on medications or treatments	FQOL—health PMD‐QOL—no discomfort due to side effects of medication, or from feeding problems San Martin Scale—prevention/treatment of problems derived from physical disabilities, sensory disabilities Brunet Lezine Scale—neurodevelopmental status
3d. Work capacity	
4. Social relationships	
4a. Personal relationships	ABC—challenging behaviour measure QSI—social impairment EMARO—% time spent on social activity FQOL—relationships QOLIP—‘Belonging’—relationships QOL‐PMD—makes wishes/needs known, has privacy, contributes to communication, uses sensory abilities, good contact with family San Martin Scale—affection and physical contact, social inclusion, communication needs and preferences respected SPOS—interpersonal relations VABS II—Receptive and expressive communication, socialisation, community
4b. Social support	FQOL—support QOL‐PMD—express preference re: support staff San Martin Scale—positive behaviour support SPOS ‐ rights
4c. Sexual activity	QOLIP—‘being’ sex QOL‐PMD—sexual needs are fulfilled
5. Environment	
5a. Physical safety and security	QOL‐PMD—lives in safe community San Martin Scale—measures to avoid falls, blows, escapes
5b. Home environment	QOLIP—‘Belonging’ home QOL‐PMD—rooms geared to their needs San Martin Scale—own space and belongings, room decorated to preferences, home environment optimised and geared to abilities
5c. Financial resources	FQOL—finances San Martin Scale—has material goods they need SPOS—material wellbeing
5d. Health & social care accessibility and quality	CSRI—service provision/access FQOL—service support QOL‐PMD—access to all needed technical aids/adaptations San Martin Scale—support to express emotion/distress/preferences/control behaviours. Staff trained on rights and ethics, rights/privacy respected, personal information kept confidential
5e. Opportunities for acquiring new information and skills	QOL‐PMD—acquires new skills/experiences San Martin Scale—opportunities to demonstrate and develop own skills, avoid over stimulation and under stimulation, have new experiences
5f. Participation in and opportunities for recreation/leisure activities	IPAQ – questions 1–4 time moderate/vigorous physical activity EMARO ‐ % time spent on audio‐visual activities, leisure activities. Sensory activities, community involvement FQOL—leisure and community QOLIP—‘belonging’—community access, ‘Becoming’—recreation and leisure QOL‐PMD—regular use of community facilities, social contacts outside carers/family/health professionals, participates in activities they can cope with & find interesting including in open air, does not have to occupy self during the day, community environment adapted to their needs. San Martin Scale—opportunity to participate or refuse, inclusion in variety of activities and community facilities SPOS—personal development, social inclusion/participation/wellbeing VABS II—play and leisure, community
5g. Physical environment (pollution/noise/traffic/climate)	
5h. Transport	
6. Spiritual/religion/personal beliefs	
	FQOL—values QOLIP—‘being’ beliefs, values, standards of conduct, hope for the future
